# Joint associations between gestational environmental chemical mixtures and child behavioral outcomes

**DOI:** 10.1016/j.envint.2026.110383

**Published:** 2026-06-25

**Authors:** Jagadeesh Puvvula, Wei-Ting Hwang, Lawrence McCandless, Changchun Xie, Joseph M. Braun, Ann M. Vuong, Enrique F. Schisterman, Russell T. Shinohara, Youssef Oulhote, Linda Booij, Jean R. Séguin, Maryse F. Bouchard, Christine Till, Angelika Zidek, Michael M. Borghese, Mandy Fisher, William D. Fraser, Gina Muckle, Kimberly Yolton, Kim M. Cecil, Jillian Ashley-Martin, Tye E. Arbuckle, Bruce Lanphear, Aimin Chen

**Affiliations:** a Department of Biostatistics, Epidemiology and Informatics, Perelman School of Medicine, University of Pennsylvania, Philadelphia, PA, USA; b Faculty of Health Sciences, Simon Fraser University, Burnaby, BC, Canada; c Department of Biostatistics, Health Informatics and Data Sciences, University of Cincinnati College of Medicine, Cincinnati, OH, USA; d Department of Epidemiology, Brown University, Providence, RI, USA; e Department of Epidemiology and Biostatistics, School of Public Health, University of Nevada, Las Vegas, Las Vegas, NV, USA; f Penn Statistics in Imaging and Visualization Center, Department of Biostatistics, Epidemiology, and Informatics, University of Pennsylvania, Philadelphia, PA, USA; g Center for AI and Data Science for Integrated Diagnostics, University of Pennsylvania, Philadelphia, PA, USA; h Department of Environmental Medicine and Public Health, Icahn School of Medicine at Mount Sinai, New York, NY, USA; i Department of Psychiatry, McGill University, Montreal, QC, Canada; j Eating Disorders Continuum, Douglas Mental Health University Institute, Montréal West Island Integrated Health and Social Services Centre, Montréal, QC, Canada; k Research Centre, Douglas Mental Health University Institute, Montréal, QC, Canada; l Azrieli Research Centre of the CHU Sainte-Justine, Montréal, QC, Canada; m Institut national de la recherche scientifique (INRS) – Centre Armand-Frappier Santé Biotechnologie, Laval, Montréal, Canada; n Psychology Department, York University, Toronto, ON, Canada; o Existing Substances and Risk Assessment Bureau, Health Canada, Ottawa, ON, Canada; p Environmental Health Science and Research Bureau, Healthy Environments and Consumer Safety Branch, Health Canada, Ottawa, ON, Canada; q Department of Obstetrics and Gynecology, Centre de Recherche du CHUS, University of Sherbrooke, Sherbrooke, QC, Canada; r CHU de Québec Research Centre and School of Psychology, Laval University, Québec City, QC, Canada; s Department of Pediatrics, Cincinnati Children’s Hospital Medical Center, University of Cincinnati College of Medicine, Cincinnati, OH, USA; t Department of Radiology, Cincinnati Children’s Hospital Medical Center, University of Cincinnati College of Medicine, Cincinnati, OH, USA; u Child and Family Research Institute, BC Children’s Hospital, Vancouver, BC, Canada

**Keywords:** Endocrine disruptors, Environmental chemicals, Gestational exposures, Child behavior

## Abstract

**Background:**

Gestational exposure to environmental chemicals contributes to adverse neurodevelopmental outcomes in children. We aim to evaluate the joint associations between gestational chemical biomarkers and behavioral functioning in preschool-aged children.

**Methods:**

We pooled data from 695 mother–child dyads (female, n = 353; male, n = 342) enrolled in the Health Outcomes and Measures of the Environment (HOME) & Maternal-Infant Research on Environmental Chemicals (MIREC) Studies. We assessed concentrations of 29 EDC biomarkers at gestation and assessed the child’s behavior and executive functioning at 3 years using the Behavioral Assessment System for Children (BASC-2) and Behavior Rating Inventory of Executive Function–Preschool Version (BRIEF-P). Higher scores on these instruments indicate adverse behavioral outcomes. We applied quantile g-computation to estimate joint associations, adjusting for covariates.

**Findings:**

Every quartile increase in chemical biomarker mixture was associated with a 0.84-point higher behavior symptom index score (95% CI: −0.89, 2.57), 1.38-point higher externalizing behavior score (95% CI: −0.65, 3.41), and 1.34-point higher internalizing behavior score (95%CI: −0.97, 3.65); these associations were imprecise with confidence intervals including null. Although the interaction by child sex remained null (p = 0.69) for internalizing behavior scores, gestational chemical mixture was associated with higher internalizing behavior scores in males (Ψ: 3.59; 95%CI: 0.42, 6.75), but not in females. We found evidence of sex-specific trends for working memory scores (interaction p = 0.02), with an imprecise positive trend in males (Ψ: 3.04; 95%CI: −0.46, 6.55) and a negative trend in females.

**Interpretation:**

Gestational exposure to a mixture of environmental chemicals showed suggestive but imprecise associations with internalizing problems and poorer working memory among preschool-age males, but not females. Associations were more pronounced in the HOME Study, which includes participants with higher concentrations for many chemical biomarkers, than in MIREC, raising the possibility that pooled estimates reflect cohort-specific exposure contexts. Sex-specific patterns, particularly for working memory, need further investigation, and our findings should be interpreted cautiously given the imprecision of subgroup estimates.

## 1. Introduction

Exposure to environmental chemicals during early brain development can have adverse effects on children, leading to inattention, hyperactivity, and impairments in behavioral, emotional, and social domains (Lanphear, 2015; Grandjean and Landrigan, 2014; Bellinger, 2013; Grandjean and Landrigan, 2006). Pregnant women are exposed to a range of chemicals, including metals, polybrominated diphenyl ethers (PBDEs), polychlorinated biphenyls (PCBs), per- and polyfluoroalkyl substances (PFAS), bisphenol A (BPA), phthalates, triclosan, organophosphate esters (OPEs), and pesticides (Woodruff et al., 2011; Buckley et al., 2022). Human exposure sources to these chemicals commonly include consumer products (OPEs, PBDEs, PFAS), personal care products (phthalates, parabens, phenols), diet (arsenic, mercury, PCBs, PFAS, BPA, pesticides), drinking water (PFAS, lead, and arsenic), and house dust (lead, PBDEs, OPEs) (Thacharodi et al., 2023; Metcalfe et al., 2022). Studies have shown that prenatal exposure to environmental chemicals is associated with behavioral problems in children (Bach et al., 2022; Caspersen et al., 2016; Colicino et al., 2021; Dewey et al., 2023; England-Mason et al., 2021; England-Mason et al., 2020; Fortenberry et al., 2014; Fruh et al., 2019; Fruh et al., 2021; Furlong et al., 2017; Harris et al., 2021; Horton et al., 2018; Ibroci et al., 2022; Leader et al., 2025; Messerlian et al., 2017; Rokoff et al., 2022; Sagiv et al., 2021; Sagiv et al., 2023; Shoaff et al., 2020; Thistle et al., 2022; Wang et al., 2025; Yang et al., 2024). Most of these studies focused on exposure to metals (Horton et al., 2018; Rokoff et al., 2022); phthalates (Colicino et al., 2021; Dewey et al., 2023; England-Mason et al., 2020; Messerlian et al., 2017; Shoaff et al., 2020); PFAS (Bach et al., 2022; Harris et al., 2021; Yang et al., 2024), parabens (Leader et al., 2025), phenols (Ibroci et al., 2022), and pesticide (Fortenberry et al., 2014; Furlong et al., 2017; Rokoff et al., 2022; Sagiv et al., 2021; Sagiv et al., 2023; Thistle et al., 2022; Wang et al., 2025) biomarkers. Overall, gestational exposure to metals (Fruh et al., 2019; Fruh et al., 2021; Horton et al., 2018; Rokoff et al., 2022), organophosphate pesticides (Sagiv et al., 2021; Sagiv et al., 2023; Thistle et al., 2022; Wang et al., 2025),pyrethroids (Furlong et al., 2017); phthalates (Colicino et al., 2021; Dewey et al., 2023; England-Mason et al., 2020; Messerlian et al., 2017; Shoaff et al., 2020; Choi et al., 2021), PFAS (Bach et al., 2022), and parabens (Leader et al., 2025) was associated with adverse behavioral outcomes among children.

We conducted literature review using the following search query (gestational OR pregnancy OR prenatal) AND (“metal*” OR “polybrominated diphenyl ether*” OR PBDE OR “polychlorinated biphenyl*” OR PCB OR “perfluoroalkyl substance*” OR PFAS OR “bisphenol*” OR phthalate* OR triclosan OR “organophosphate ester*” OR pesticide* OR paraben* OR phenol*) AND (“Behavior Assessment System for Children” OR “Behavior Rating Inventory of Executive Function”) AND humans [MeSH Terms] AND English [Language], on PubMed on June 02, 2025. Among the 38 original research articles retrieved from our search query, we excluded 12 studies that include participants from the existing work (the HOME and MIREC Studies) to avoid duplication and a study that was retracted. The remaining 26 peer-reviewed articles included participants from birth cohorts across the US, Canada, Mexico, Europe, and China. Since humans are concurrently exposed to a multitude of environmental chemicals, it is critical to examine additive or synergistic associations between prenatal environmental chemical biomarkers and child behaviors (Carlin et al., 2013). Additionally, unmeasured exposures may produce biased estimates. Few studies have examined distinct classes of endocrine-disrupting chemicals (EDCs) as a chemical mixture (10,24,27,29)**. To address this potential gap, we assessed the joint associations between 29 chemical biomarkers frequently detected during early gestation and preschool children’s behaviors by pooling study participants from two prospective North American birth cohorts.

## 2. Methods

### 2.1. Study population

This study includes mother–child dyads enrolled in two prospective birth cohorts in North America: the Health Outcomes and Measures of the Environment (HOME) Study (Braun et al., 2017), based in the Cincinnati, Ohio area, and the Maternal-Infant Research on Environmental Chemicals (MIREC) Study (Arbuckle et al., 2013), which includes participants from 10 cities across Canada.

### 2.2. HOME study

Study participants included women receiving care at prenatal practices affiliated with three hospitals between 2003 and 2006 (Braun et al., 2017). Women were eligible if they were 16 ± 3 weeks pregnant, ≥18 years old, residing in a home built in or before 1978, not living in a mobile or trailer home, HIV-negative, not taking medications for seizures or thyroid disorders, planning to continue prenatal care and deliver at the collaborating clinics and hospitals, planning to live in the greater Cincinnati area for the next year, fluent in English, and without a diagnosis of diabetes, bipolar disorder, schizophrenia or cancer that resulted in radiation treatment or chemotherapy (n = 1,263). Among the eligible participants, 468 women were enrolled and completed the first study visit at 16 weeks (range 13–19) of gestation. Among those women who delivered a live singleton newborn, 389 mother–child dyads were considered for follow-up.

### 2.3. MIREC study

The MIREC study recruited pregnant women from prenatal clinics with established clinical obstetrical research infrastructure across 10 Canadian cities (ranging from Vancouver, BC, to Halifax, NS) between 2008 and 2011 (Arbuckle et al., 2013). Women aged at least 18 years in their first trimester (6–13 weeks of gestation), without known fetal abnormalities, without a history of renal/heart/serious pulmonary diseases, epilepsy, collagen disease, active/chronic hepatic diseases, cancer, hematological disorder, threatened spontaneous abortion, and illicit drug use were invited for enrollment (n = 5108). Among these potential study participants, 2001 consented to participate. Of the enrolled participants, 1983 completed the first visit (6–13 weeks of gestation) and were considered for follow-up.

### 2.4. Environmental chemical exposure biomarkers

Biospecimens (whole blood, serum, and urine) from the participants enrolled in the HOME and MIREC Studies were collected at their first clinical visit to measure biomarkers of environmental chemical exposures, except for urinary arsenic in the HOME Study, with 94% at the second (~26 weeks of gestation) clinical visit. We measured concentrations of 44 biomarkers among participants in both studies, and 29 biomarkers detected in ≥50% of participants were included in our analysis (eFigs. 1 & 2).

Biomarkers with lipophilic properties and longer biological half-lives were measured in serum or plasma samples. In contrast, those with hydrophilic properties and shorter biological half-life were measured in urine samples. Most biomarkers (except dimethylarsinate [DMA], measured at Dartmouth College) from the biospecimens collected from HOME Study participants were measured at the Centers for Disease Control and Prevention (CDC, Atlanta, GA). All chemical biomarkers from the MIREC study were measured at the Institut National de Santé Publique du Québec (INSPQ, Québec, Canada). Analytical methods for biomarker quantification, imputing values below the LOD, and biomarker standardization strategies are described in the supplementary material (eMethods 1).

### 2.5. Imputing biomarker measurements

Among the 29 chemical biomarkers detected in >50% of the samples, the values below the LOD were imputed using the left-truncated lognormal distribution for each analyte through the “fill-in” method (Lubin et al., 2004; Mersmann et al., 2023).

### 2.6. Standardization of biomarker measurements

Serum biomarker (PBDEs, PCBs) concentrations were standardized using the serum lipid measurements, as outlined in equation (1). In the HOME Study, total lipid concentrations were calculated using the short form of the Philips formula: (2.27 * TC) + TG + 62.3, where TC refers to serum total cholesterol and TG refers to triglycerides (Phillips et al., 1989). The MIREC Study estimated the total lipid value using 1.677* (TC-FC) + FC + TG + PL (Patterson et al., 1991). The biomarker concentrations from urine samples were standardized using urine specific gravity, following Eq. (2) (O’Brien et al., 2016; Hauser et al., 2004).


(1) $${\text{Concentration}}_{\text{lipidstandardixed}}=\frac{\left({\text{Biomarkerconcentration}}_{i}\right)}{\text{Totallipid}}\ldots$$

(2) $${\text{Concentration}}_{\text{urine-specificgravitystandardixed}}={\text{concentration}}_{i}\frac{\left(SG_{m}-1\right)}{\left(SG_{i}-1\right)}\ldots$$

*SG_i_* = *individual specific gravity measurement & SG_m_* = *median specific gravity measurement*.

We aggregated phthalate metabolites using the molar sum of MEHHP (mono-2-ethyl-5-hydroxyhexyl phthalate), MEHP (mono-2-ethylhexyl phthalate), MECPP (mono-2-ethyl-5-carboxypentyl phthalate), and MEOHP (mono-2-ethyl-5-oxohexyl phthalate) to represent exposure to di(2-ethylhexyl) phthalate (DEHP). Additionally, organophosphate insecticides were aggregated to diethylphosphate (DEP = molar sum of diethylphosphate and diethylthiophosphate) and dimethylphosphate (DMP = molar sum of dimethylphosphate and dimethylthiophosphate). Individual metabolites of DEHP, DEP, and DMP were excluded from further analysis.

### 2.7. Child behavioral assessments

The MIREC Study’s neurodevelopmental assessments were conducted among participants in the CD Plus phase, which included 896 participants from the six most populous recruitment sites (Fisher et al., 2023). Among the MIREC Study participants, 12 have opted out of the biobank and were excluded from our study. We assessed child behaviors at ~ 3 years of age in the HOME Study (n = 247) and between 3 and 4 years in the MIREC Study (n = 884). The analysis was restricted to singleton births. Parent-reported adaptive and problem behavior scores were measured using the Behavioral Assessment System for Children (BASC-2) (Reynolds, 2004). Additionally, children’s executive function was assessed using two subscales of the Behavior Rating Inventory of Executive Function (BRIEF) parent form (Gioia et al., 2000).

#### 2.7.1. Behavioral assessment

The BASC-2 assessment provides three composite scores: Behavioral Symptom Index (BASC-BSI), Externalizing Problems (BASC-EXT), and Internalizing Problems (BASC-INT), which are available in both HOME and MIREC studies. The BSI scores indicate hyperactivity, aggression, depression, atypicality, withdrawal, and attention problem-related attributes; EXT scores indicate hyperactivity, aggression, and conduct problems; and INT indicates anxiety, depression, and somatization. Behavioral assessment scores were standardized using the publisher-provided age-specific norms, yielding a mean of 50 and a standard deviation of 10. Higher behavioral scores indicate more non-optimal behaviors.

#### 2.7.2. Executive function

Two subscales of the BRIEF-P, available in both the HOME and MIREC studies, were included in the analysis: planning and organizing (BRIEF-PO) and working memory (BRIEF-WM). The PO subscale measures difficulties in preparing for future events, and WM measures difficulties in retaining information needed to complete a task. Raw scores were converted to age- and sex-normalized T-scores, with a mean of 50 and a standard deviation of 10. A higher score indicates poorer planning or working memory skills.

### 2.8. Covariates

We identified maternal and child-related covariates for this study from the literature using a directed acyclic graph (eFig. 3).

#### 2.8.1. Maternal factors

We included maternal age, race, education, parity, and cotinine concentrations (log_2_-transformed). Participants with cotinine concentrations below the LOD were imputed using the “fill-in” method following a left-truncated lognormal distribution (Lubin et al., 2004; Mersmann et al., 2023). Maternal race was coded into two categories: White and other. Maternal education was coded into three levels: less than or equal to high school education, greater than high school and less than postgraduate education, and postgraduate or higher education. Two participants from the MIREC study with missing maternal education were imputed to the dominant category (greater than high school and less than a postgraduate education). Additionally, we included maternal depression measured during the child’s 3-year visit as a potential covariate. In the HOME Study, maternal depression was measured using the Beck Depression Inventory-II (BDI-II) (Beck et al., 1996), a 21-item questionnaire that captures Likert-scale responses, yielding scores ranging from 0 to 63. Whereas, in the MIREC Study, maternal depression was measured using the Center for Epidemiologic Studies Depression (CES-D) scale (Radloff, 1977), a 20-item checklist. Maternal depression scores were transformed to a z-score scale independently by cohort.

#### 2.8.2. Child-related factors

We included child sex and the Home Observation for Measurement of the Environment (HOME) score, a proxy for the caregiving environment. These scores were assessed at 1 year in the HOME Study and at 3 years in the MIREC Study (Bradley and Caldwell, 1980). There are 100 participants with unmeasured HOME scores from the MIREC Study, which were imputed using the SuperLearner algorithm (Mean Squared Error = 19.95) (Polley et al., 2021).

Additionally, we adjusted for city and cohort to account for potential geographic heterogeneity.

### 2.9. Statistical analysis

Analyses were performed using 695 mother–child dyads, with measurements of 29 chemical biomarkers and child behavioral assessments, comprising 139 from the HOME Study (female, n = 73; male, n = 66) and 556 from the MIREC Study (female, n = 280; male, n = 276) (eFig. 2).

#### 2.9.1. Joint associations using quantile g-computation

We examined joint associations between 29 gestational chemical biomarkers, considered as a mixture, and child behavioral assessments using quantile g-computation regression (Keil et al., 2020). The model was fit, assuming a normal distribution of behavioral outcomes and considering chemical biomarkers on a quartile scale. A detailed model specification is outlined in the supplemental material. This model also includes adjustments for the previously mentioned covariates. We performed the non-bootstrap variant of the quantile g-computation regression to obtain the overall effect (*Ψ*), scaled positive effect (+*Ψ*), scaled negative effect (−*Ψ*), and the chemical biomarker weights that contributed to the joint association. We multiplied the chemical biomarker weights by −1 for those that contributed negatively to the associations for presentation purposes. We emphasized chemical biomarkers with weights exceeding a specific threshold (2/29). Additionally, we performed the bootstrap variant of the quantile g-computation regression using 400 bootstrap iterations to highlight the potential uncertainty of estimates from the non-bootstrap model.

Furthermore, we explored the role of child sex as a potential effect measure modifier and reported p-values for the interaction term using the quantile g-computation method. In addition to the overall analysis, we presented the results stratified by child sex. This analysis was performed using the *qgcomp* and *qgcompint* R packages (Keil, 2022; Keil, 2022).

#### 2.9.2. Single chemical biomarker associations using linear regression

We used multivariable linear regression to compare the effect estimates from the quantile g-computation with those from individual chemical biomarkers, including 695 participants with all 29 chemical biomarkers. Additionally, we analyzed the associations using up to 894 study participants (HOME = 243 & MIREC = 651) with data on at least one chemical biomarker, outcome, and covariate, to maximize power. This single chemical analysis was extended to the study site level to examine potential heterogeneity. Chemical biomarker concentrations were transformed to a log_2_ scale. The effect estimates (β) correspond to a change in behavioral test scores for every 2-fold increase in chemical biomarker concentrations.

#### 2.9.3. Sensitivity analysis

Since 15% of the study participants in our analytical sample have unmeasured caregiving environment scores, we performed multiple imputations using the chained equations algorithm, generating 15 imputed datasets (Buuren and Groothuis-Oudshoorn, 2011). Along with the joint associations assessed using single imputations, we supplemented the findings from the multiple-imputed data to highlight potential uncertainty due to unmeasured caregiving environment scores. We performed bootstrap and non-bootstrap variants of quantile g-computation regression across 15 multiple imputed datasets and pooled the estimates using Rubin’s rule (Buuren and Groothuis-Oudshoorn, 2011). Furthermore, we evaluated joint associations using singly imputed data, following the quantile g-computation approach by study sites to observe potential heterogeneity. All analyses were performed using R version 3.2 statistical software (R Foundation for Statistical Computing) (R: A Language and Environment for Statistical Computing, 2026).

This work complies with the Strengthening the Reporting of Observational Studies in Epidemiology (STROBE) guidelines for cohort studies (Vandenbroucke et al., 2007a,b, 2014). The completed STROBE checklist is available in Appendix 1.

### 2.10. Role of the funding source

The funder had no role in the study design, data collection, data analysis, data interpretation, or writing of the report.

## 3. Results

### 3.1. Participant characteristics

The median age of women at delivery was 33 years [interquartile range (IQR): 29.43, 36.00]; 85.76% were White, similar to the overall cohort (eTable 2). Most (60%) mothers completed at least a high school education, and fewer than 10% completed postgraduate education. About half (53%) of the women had detectable cotinine levels. Forty-four percent of women delivered their first newborn. Half of the children included in this study (50.79%) were females.

### 3.2. Gestational exposure biomarkers and child behavioral assessment trends

**Fig. 1.**
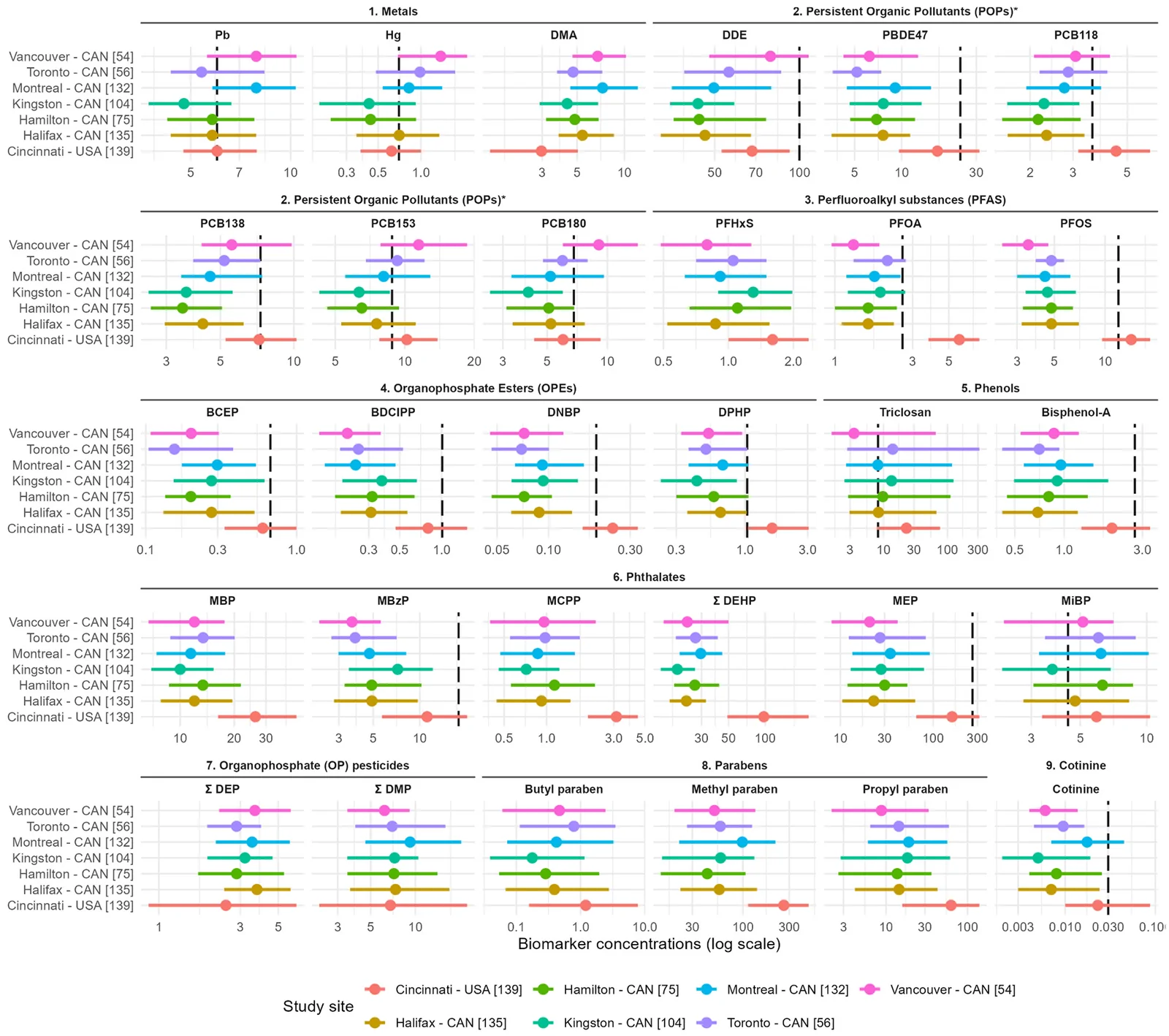
Gestational chemical biomarker summary by study site. Vertical dashed line represents the median concentration from the National Health and Nutrition Examination Survey pregnant population (for biomarkers except for OPEs)^5^ and median concentrations for OPEs obtained from the Environmental influences on Child Health Outcomes^57^ pregnant population.

Among the participants in this study, certain biomarkers of OP pesticides and OPEs had lower detection frequencies than those of other chemical biomarker classes (eTable 1). In the HOME Study, participants had higher PFAS, OPEs, phthalates, parabens, and phenol biomarker concentrations than those in the MIREC Study (Table 1). Although exposure biomarker concentrations were relatively higher among the HOME Study participants, these patterns align with those observed in the pregnant population enrolled in the US National Health and Nutrition Examination Survey (Woodruff et al., 2011) and the Environmental influences on Child Health Outcomes cohort (Oh et al., 2025). Among the MIREC Study participants, exposure biomarkers showed heterogeneity by the study site. For example, participants from the Vancouver site had higher concentrations of metals and POPs, whereas participants from Hamilton had higher concentrations of PFAS biomarkers (Fig. 1). Biomarkers of PCB congeners showed relatively strong interclass correlations, whereas other biomarkers showed weak to moderate correlations (eFig. 4). Child behavioral assessment scores were similar among children in both cohorts (Table 1).

### 3.3. Joint associations between gestational chemical biomarkers and child behavior

We observed positive trends between gestational chemical biomarkers and child behaviors (eFig. 5; Table 2), including Behavioral Symptom Index (*Ψ*: 0.84; 95% CI: −0.89, 2.57), Externalizing (*Ψ*: 1.38; 95% CI: −0.65, 3.41), and Internalizing (*Ψ*: 1.34; 95% CI: −0.97, 3.65) Problems, although confidence intervals included the null for all three outcomes. While the effect measure modification by child sex remained null (p-value > 0.1), we observed diverging trends for Internalizing Problems when stratifying the analysis by child sex. Among males, a one-quartile increase in gestational chemical biomarkers was associated with a 3.59-point increase (95% CI: 0.42, 6.75) in Internalizing Problems scores, whereas in girls, this association remained null (*Ψ*: −0.89; 95% CI: −4.07, 2.30). The association among males was primarily driven by PCB180, Σ DEHP, and methyl paraben (eFig. 6).

### 3.4. Joint associations between gestational chemical biomarkers and child executive function

We observed an overall null association between gestational chemical biomarkers and child executive function (*Ψ_BRIEF-PO_*: 0.31; 95% CI: −2.11, 2.73 & *Ψ_BRIEF-WM_*: −0.38; 95% CI: −3.01, 2.25). We observed a statistically significant effect modification by child sex (p-value_EMM_ = 0.02) in the association between the gestational chemical biomarker mixture and working memory. Among males, a one-quartile increase in gestational chemical biomarkers was associated with a 3.04-point increase (95% CI: −0.46, 6.55) in working memory score, although this difference was not statistically significant. This positive trend among males was primarily driven by chemical biomarkers of PBDE47, PCB153, BCEP, BPA, MEP, and ΣDEP. In contrast, among girls, every quartile increase in gestational chemical biomarker mixture was associated with a 3.40-point decrease (95% CI: −7.33, 0.53) in working memory score; this trend was primarily driven by PCB180 and MEP.

### 3.5. Sensitivity analysis

#### 3.5.1. Single chemical biomarker associations

**Fig. 2.**
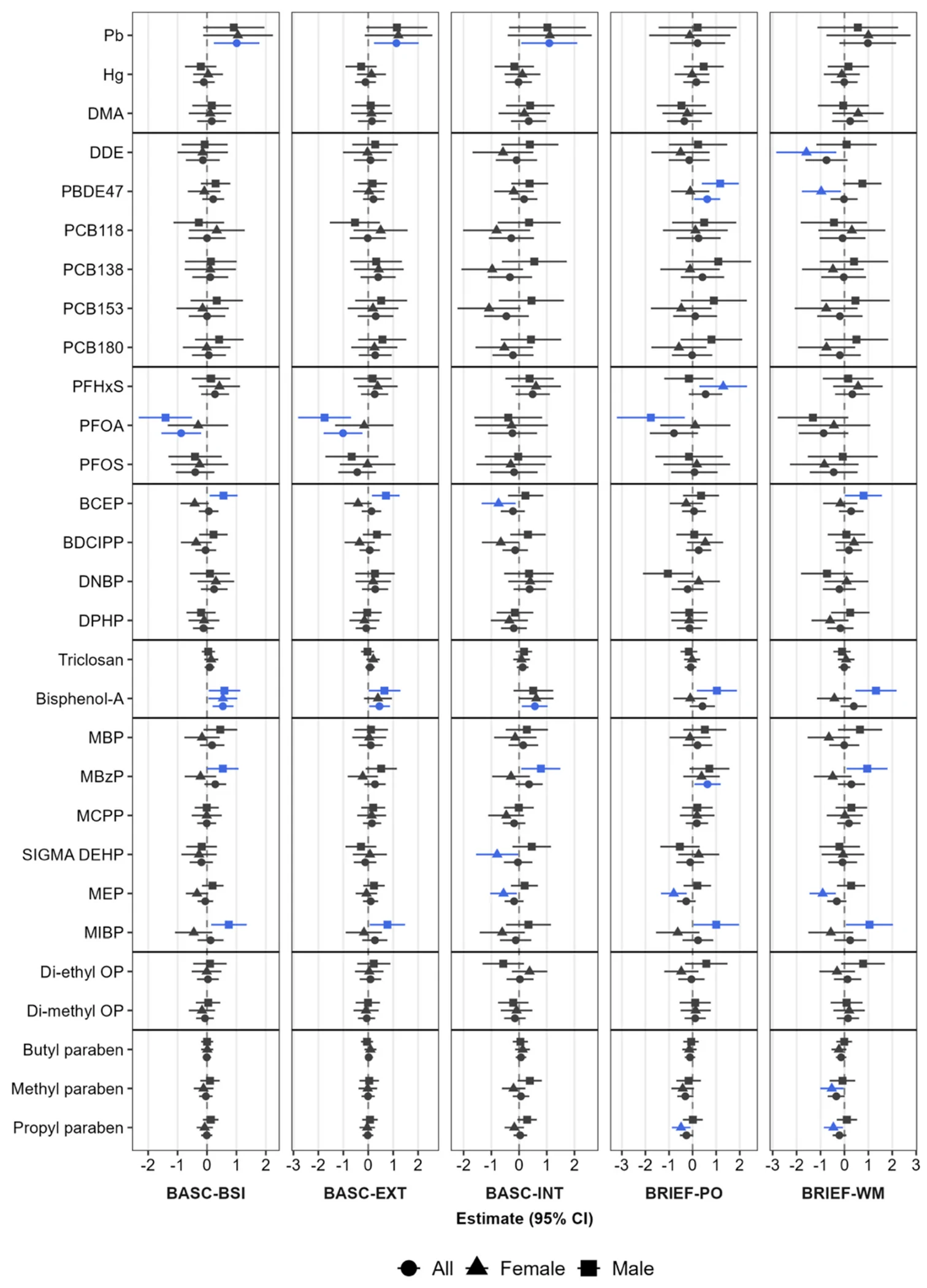
Single gestational chemical biomarker associations with child behavioral outcomes (n = 695; females = 353, males = 342). Effect estimates and 95% confidence intervals generated using multiple linear regression and interpreted as every two fold increase in gestational exposure biomarker is associated with child behavioral outcome. Confidence intervals not covering the null were highlighted in blue. Refer Table S4 for numeric values of estimates and 95% CIs. (For interpretation of the references to colour in this figure legend, the reader is referred to the web version of this article.)

We observed that gestational biomarkers of Pb and BPA were associated with higher Behavioral Symptom Index scores (Fig. 2, eTable 3 & eFig. 7). We observed similar patterns when analyzing single-chemical biomarker associations while using a subset of participants with all 29 chemical biomarkers (n = 695) compared to the participant set with varying exposure measurements that included up to 894 participants (eFig. 8). Additionally, gestational biomarkers of PBDE47 (β: 0.62; 95% CI: 0.07, 1.17) and MBzP (β: 0.63; 95% CI: 0.08, 1.19) were associated with higher scores in planning and organizing. While analyzing these associations stratified by child sex, the associations between biomarkers PCB180, ΣDEHP, and methyl paraben showed a positive trend with Internalizing Problems among males, but did not reach statistical significance. From the single chemical results, every two-fold increase in MBzP was associated with a 0.79-point increase (95% CI: 0.08, 1.49) in Internalizing Problems among males. We observed that BCEP (β: 0.8; 95% CI: 0.02, 1.57) and BPA (β: 1.32; 95% CI: 0.46, 2.18) were associated with increased working memory score among males, along with MBzP (β: 0.95; 95% CI: 0.09, 1.80) and MiBP (β: 1.05; 95% CI: 0.06, 2.03). Additionally, we observed heterogenous diverging patterns in single chemical biomarker exposure–response associations when stratified by MIREC Study sites. For example, every two fold increase in gestational lead concentrations among participants from the Kingston study site (n = 127) showed 2.29 point increase (95%CI: 0.54, 4.04) in BSI scores, compared to participants from the Vancouver site (n = 66) showed null associations.

#### 3.5.2. Joint associations using multiple imputations for the HOME score

The joint-effect estimates remained similar when pooling results from 15 imputed datasets, compared with those from a single imputation of the caregiving environment scores (eTable 4). The effect estimates were altered up to ±0.12 without altering the direction of association trends. While analyzing these associations stratified by child sex, the effect estimates varied up to ±0.26 without altering the direction of association trends.

#### 3.5.3. Joint associations stratified by cohort

For the joint associations stratified by cohort, a distinct pattern was observed (eTable 5). In the HOME Study, each quartile increase in gestational exposure to chemical biomarker mixtures was associated with a 5-point increase in Behavioral Symptom Index score (95% CI: −1.62, 11.63), whereas the MIREC Study found a 0.06-point decline (95% CI: −1.65, 1.52) in BSI scores. Every quartile increase in gestational exposure mixtures was associated with a 7.13-point increase (95% CI: −0.15, 14.41) in Externalizing Problems in the HOME Study, compared to a 0.66-point increase (95% CI: −1.32, 2.64) in the MIREC Study. Given that HOME Study participants had higher exposure concentrations across the majority of biomarkers (Section 3.2), these patterns suggest that the pooled positive point estimates are weighted toward the higher-exposure cohort and may not represent associations that generalize across exposure contexts.

Furthermore, we extended this analysis stratified by study site and observed heterogeneous joint association patterns within the MIREC Study sites (eFig. 9). Among the BASC outcomes, we observed joint associations among participants from the Vancouver site (n = 54) that aligned with those observed in the HOME Study participants. In contrast, we observed associations between the 29-chemical biomarker mixture and improved BRIEF scores in 132 participants from the Montreal study site.

## 4. Discussion

### 4.1. Summary of current findings

In our pooled analysis, we observed positive trend effect estimates for behavioral scores (BSI, EXT, INT) associated with the gestational chemical mixture, although the 95% confidence intervals included the null value. Effect modification by child sex was not statistically significant; however, findings stratified by child sex showed that the gestational chemical mixture was associated with increased internalizing scores among males, driven by PCB180, ΣDEHP, and methyl paraben, whereas null trends were observed among girls. Associations between gestational chemical mixture and child executive function remained null. Still, effect modification by child sex was statistically significant for working memory scores (p = 0.02), with a positive association in males and an inverse association in girls. The positive associations with working memory scores in males were driven by PBDEs, BCEP, BPA, MEP, and ΣDEP. Sensitivity analyses revealed associations between individual biomarkers (e.g., lead, BPA, PBDE47, MBzP) and increased scores for behavioral problems or executive function. In males, MBzP, MIBP, and BCEP were linked with higher internalizing and working memory scores. Importantly, we found stronger associations with BSI and EXT in the HOME Study than in the MIREC Study, highlighting cohort-specific effects.

While stratifying by study sites, we observed diverging exposure–response association patterns. Although the sample size was relatively small at the Vancouver study site, an increase in chemical biomarker mixtures was associated with worse BASC scores (BSI & EXT), while estimates at the other study sites remained null. In Montreal, we observed increase in chemical mixtures was associated with improved BRIEF scores, compared to the other sites that showed null patterns. Heterogeneity of the association patterns could be multifaceted, possibly due to (1) distinct exposure–response curves observed at varying exposure ranges; (2) residual underlying socio-economic differences that drive protective behavior; (3) insufficient consideration of additional unmeasured exposures/protective factors.

### 4.2. Findings in the context of previous literature

From our literature review, we identified that a majority of the studies were based in the US-based cohorts (Viva (Fruh et al., 2019; Fruh et al., 2021; Harris et al., 2021), CEHC (Furlong et al., 2017), TIDES (Ibroci et al., 2022), PACE (Leader et al., 2025), EARTH (Messerlian et al., 2017); NBC (Rokoff et al., 2022; Shoaff et al., 2020); CHAMACOS (Sagiv et al., 2021; Sagiv et al., 2023), and a few studies from Canada (APrON (Dewey et al., 2023; England-Mason et al., 2021; England-Mason et al., 2020), Mexico (PROGRESS (Colicino et al., 2021); ELEMENT (Fortenberry et al., 2014; Horton et al., 2018), Europe (MoBa (Caspersen et al., 2016; Thistle et al., 2022), DNBC (Bach et al., 2022), and China (SBC (Wang et al., 2025; Yang et al., 2024). Overall, these studies reported associations between bisphenol-A, phthalates, and child behavioral outcomes, where the associations showed diverging patterns by child sex. Although there is extensive evidence linking gestational EDCs with child behavior, few studies (Rokoff et al., 2022; Wang et al., 2025; Vuong et al., 2016) have examined distinct classes of exposures as a mixture. Because humans are typically exposed to multiple chemical compounds, our findings uncover potential joint associations between distinct classes of EDCs and child behavior.

Prenatal blood lead levels consistently contributed positively to the joint associations between gestational exposure mixtures and child behaviors. Similar to our findings, Project Viva reported associations between prenatal blood lead concentrations and parent-rated BRIEF plan/organize scores (Fruh et al., 2019), as well as patterns linking lead to worse neurobehavioral scores (Fruh et al., 2021) when metals were considered as a mixture. In the ELEMENT project, dentine-based reconstructions showed postnatal lead concentrations were associated with higher BSI scores, externalizing problems, and anxiety symptoms (Horton et al., 2018). The NBC study also found positive trends between prenatal lead exposure and anxiety/internalizing problems in both males and females^24^, aligning with our single-chemical analyses.

Prenatal BPA exposures in our study were consistently associated with behavioral problems, except for BRIEF outcomes among girls. In contrast, the TIDES study, which involved participants with relatively high socioeconomic status, reported nonsignificant trends between prenatal BPA exposure and BASC-2 scores in females (Ibroci et al., 2022). Among the phthalates, MiBP was associated with higher BASC/BRIEF scores (except internalizing) in males, whereas MBzP was associated with lower BASC/BRIEF scores (except externalizing). Although MCPP, ΣDEHP, MEP, and MiBP generally showed null associations, sexually dimorphic patterns were evident. Consistent with our findings, the EARTH Study reported associations between gestational MiBP and BASC BSI/externalizing scores in males (Messerlian et al., 2017), and the MoBa study linked MBzP to parent-reported working memory scores in males (Choi et al., 2021). For OPEs, gestational BCEP was associated with BASC (BSI & EXT) and BRIEF working memory scores in males. In contrast, the SBC study examined DPHP alone and found null associations with internalizing or externalizing scores (Wang et al., 2025).

In our analysis, gestational PFHxS was associated with females’ BRIEF plan/organize scores, whereas PFOA and PFOS showed null or protective trends. Similarly, the SBC study (Yang et al., 2024) and Project Viva (Harris et al., 2021), reported null associations between gestational PFAS exposures and BRIEF scores. We observed associations between PBDE47 and BRIEF outcomes in males, while the GESTE study reported null associations (Solazzo et al., 2021). Although organophosphate pesticides showed null associations in our study, CHAMACOS reported links between DAP and executive function (Sagiv et al., 2021), internalizing/externalizing scores (Sagiv et al., 2023), and the MoBa study associated DEP/DMP with working memory (Thistle et al., 2022) among children. The potential mechanism was detailed in the supplement.

### 4.3. Potential mechanistic interpretation

Exposure to EDCs is known to interfere with hormone systems, such as acting as nuclear receptor agonists or antagonists and disrupting hormone synthesis, transport, distribution, or clearance (La Merrill et al., 2020). Alterations *in* hormones during critical developmental windows, such as gestation, whe*n* the placenta is abundant *in* endocrine receptors, may influence fetal neurodevelopment (Schug et al., 2015; Gingrich et al., 2020). Findings from pre-clinical studies suggest probable associations between exposure to BPA and phthalates and behavioral abnormalities (Bakoyiannis et al., 2021; Repouskou et al., 2020; Patisaul and Adewale, 2009). EDCs may disrupt the hypothalamic-pituitary–gonadal, hypothalamic-pituitary-thyroid, and hypothalamic–pituitary–adrenal axes, altering steroid hormone (estrogen, androgen, thyroid, and glucocorticoid) signaling, neurotransmission (dopamine, serotonin, glutamate, gamma-aminobutyric acid, and acetylcholine), and affecting neurodevelopment, potentially leading to sex-specific behavioral changes (Schug et al., 2015; Repouskou et al., 2020; Ramirez et al., 2022; Patisaul et al., 2021; Batista and Hensch, 2019).

### 4.4. Strengths and limitations

We were able to pool data from two prospective pregnancy cohorts in North America with diverse exposure patterns and distinct sociodemographic characteristics to improve generalizability compared to single-site studies. While pooling participants from two studies increased our sample size and enabled us to produce more stable estimates, our site-specific analyses indicated heterogeneous exposure-outcome associations. This resulted in non-transportable estimates; our findings suggest that pooled estimates should be interpreted alongside the site-specific heterogeneity we observed. Although both studies include robust measures of potential confounders, the overall heterogeneity of our findings underscores plausible unmeasured confounding by study site. Additionally, including chemical biomarkers commonly detected in the study populations may improve the relevance of our findings and improve risk assessments in environmental health policy-making efforts.

Our study has some limitations. Excluding chemical biomarkers that were not detected in most of the study population may introduce bias in the selection of exposure variables. The inclusion of exposure biomarkers with a relatively short biological half-life, measured only once during pregnancy, may introduce exposure misclassification bias. The joint associations estimated using the quantile g-computation approach are sensitive to multicollinearity; therefore, we supplemented our results with single-chemical biomarker associations. Single imputation was performed for ~15% of participants with missing HOME scores, a covariate, potentially introducing confounder misclassification bias. To address this, we supplemented our joint association results with single imputations, pooling estimates across 15 imputed datasets. Another limitation is that, although we included 29 chemical biomarkers, our analysis was limited to participants with measurements for all chemical biomarkers. This reduction in sample size may increase the risk of false negatives (Type II errors), potentially missing exposure–response effect sizes that often require larger samples to detect. Additionally, we addressed the potential selection bias by comparing the characteristics of the subset of participants with those of the complete cohort; the two groups were similar, suggesting minimal or no bias in our findings. To evaluate potential selection bias from our exposure assessment criteria, we conducted a sensitivity analysis using the maximum available sample of participants with at least one measured chemical biomarker (n ~ 894) and found similar results with those from the restricted analytic subset (n = 695) used in our primary analysis.

## 5. Conclusion

In this pooled analysis of two North American pregnancy cohorts, gestational exposure to a 29-biomarker chemical mixture showed suggestive but imprecise associations with behavioral problems and executive function in preschool-age children. Pooled estimates for the behavior symptom index, externalizing, and internalizing problems had confidence intervals that included the null. Cohort-stratified analyses indicated that these positive trends were driven primarily by the HOME Study, where biomarker concentrations were relatively higher than in MIREC, and observed heterogeneous exposure–response patterns across the MIREC Study sites. Although the interaction by child sex was not statistically significant for internalizing problems, we observed that gestational EDC exposures were associated with worsened internalizing behaviors in males, where PCB180, ΣDEHP, and methyl paraben primarily contributed to the associations. For working memory, we observed a statistically significant sex interaction with opposing directions of association in males and females; the male-specific estimate was positive but imprecise, where biomarkers of PBDE47, PCB153, BCEP, BPA, MEP, and ΣDEP contributed to the joint associations. These findings should be interpreted in the context of substantial cohort and site-level heterogeneity in chemical exposure. Replication in cohorts with diverse exposure distributions is needed to establish the reproducibility of our findings.

## Supplementary Material

1

2

## Figures and Tables

**Table 1:** Median (25th and 75th percentile) of gestational chemical biomarkers and child behavioral assessment scores among study participants.

	Pooled (n =695)	HOME (n =139)	MIREC (n =556)
**Biomarker**			
Pb	6.22 (4.56,8.40)	6.00 (4.75, 7.90)	6.22 (4.56, 8.49)
Hg	0.68 (0.36, 1.31)	0.62 (0.38, 1.00)	0.70 (0.34, 1.38)
DMA	5.05 (3.19, 7.89)	2.96 (1.40, 5.05)	5.53 (3.71, 9.01)
DDE	53.97 (37.92,80.42)	67.8 (52.85,92.35)	49.07 (35.68,76.98)
PBDE47	8.54 (4.71, 15.92)	16.9 (9.60, 31.35)	7.27 (4.21, 12.25)
PCB118	2.80 (1.90, 4.26)	4.50 (3.15, 6.20)	2.50 (1.77, 3.55)
PCB138	5.00 (3.28, 7.42)	7.20 (5.25, 10.25)	4.44 (3.00, 6.56)
PCB153	8.26 (5.52, 12.38)	10.2 (7.75, 13.85)	7.62 (5.13, 11.74)
PCB180	5.4 (3.59, 8.52)	6 (4.30, 9.25)	5.27 (3.33, 8.41)
PFHxS	1.10 (0.68, 1.80)	1.60 (1.00, 2.35)	0.99 (0.65, 1.6)
PFOA	2.00 (1.20, 3.4)	5.80 (3.75, 7.70)	1.70 (1.10, 2.50)
PFOS	5.20 (3.50, 8.80)	14.30 (9.60, 18.50)	4.55 (3.20, 6.30)
BCEP	0.29 (0.15, 0.64)	0.60 (0.33, 1.00)	0.24 (0.14, 0.51)
BDCIPP	0.36 (0.19, 0.71)	0.8 (0.47, 1.52)	0.29 (0.16, 0.55)
DNBP	0.10 (0.06, 0.18)	0.24 (0.16, 0.33)	0.08 (0.05, 0.14)
DPHP	0.72 (0.38, 1.35)	1.61 (1.05, 3.05)	0.58 (0.32, 1.01)
TCS	11.34 (3.02, 105.02)	22.86 (7.87, 76.69)	8.93 (2.45, 110.72)
BPA	0.95 (0.54, 1.78)	1.96 (1.27, 3.34)	0.8 (0.48, 1.37)
MBP	13.14 (8.21, 23.72)	26.18 (16.24, 44.32)	11.62 (7.60, 18.83)
MBzP	5.50 (3.12, 11.18)	11.14 (5.69, 20.22)	4.80 (2.93, 9.13)
MCPP	1.13 (0.56, 2.33)	3.13 (1.97, 4.44)	0.89 (0.48, 1.66)
Σ DEHP	28.01 (17.83, 56.04)	97.53 (48.88, 227.81)	24.33 (16.55, 36.1)
MEP	37.71 (13.86, 115.47)	159.84 (65.75, 314.84)	26.22 (12, 67.34)
MiBP	5.39 (2.96, 8.93)	5.92 (3.36, 10.36)	5.23 (2.92, 8.50)
Σ Di-ethyl organophosphate	3.34 (1.85, 5.54)	2.47 (0.87, 6.39)	3.39 (2.01, 5.42)
Σ Di-methyl organophosphate	7.48 (3.41, 15.84)	6.79 (2.25, 22.33)	7.51 (3.68, 14.34)
Butyl paraben	0.42 (0.07, 3.46)	1.2 (0.16, 7.79)	0.32 (0.05, 2.55)
Methyl paraben	78 (24.88, 209.77)	264 (112.92, 475.96)	60.21 (19.05, 145.81)
Propyl paraben	19.93 (4.78, 70.46)	62.93 (15.81, 139.92)	15.6 (3.42, 50.5)
**Behavior Outcomes**			
BASC-BSI	50 (46, 55)	50 (44.5, 55)	50 (46, 55)
BASC-EXT	48 (44, 54)	48 (44, 55)	49 (44, 54)
BASC-INT	51 (45, 57)	49 (42.5, 55)	51 (46, 57)
BRIEF-PO	51 (44, 58)	51 (41, 58)	51 (44, 58)
BRIEF-WM	52 (45, 60)	53 (45, 62)	52 (45, 58)

The above table summarizes using the median and the interquartile ranges of the biomarkers and outcomes included in this study. Above chemical biomarkers were measured as ng/mL, except POPs measured as ng/g of lipid. Chemical biomarkers measured in urine were standardized for urine specific gravity. Refer Fig. 1. for study site specific exposure biomarker summaries.

**Table 2:** Associations between 29 chemical biomarkers and child behavioral outcomes using the quantile g-computation method (n = 695; female = 353, male = 342).

	BASC									BRIEF					
	BSI			EXT			INT			PO			WM		
	All	Female	Male	All	Female	Male	All	Female	Male	All	Female	Male	All	Female	Male
Overall bootstrap effect (Ψ, 95% CI)	0.84(−0.89, 2.57)	−0.19 (−2.90, 2.53)	1.79 (−0.69, 4.27)	1.38 (−0.65, 3.41)	0.87 (−2.30, 4.03)	2.07 (−0.86, 5.00)	1.34 (−0.97, 3.65)	−0.89 (−4.07, 2.30)	3.59 (0.42, 6.75)	0.31 (−2.11, 2.73)	−0.75 (−4.66, 3.17)	2.00 (−1.41, 5.41)	−0.38 (−3.01, 2.25)	−3.40 (−7.33, 0.53)	3.04 (−0.46, 6.55)
Child sex-effect modifier p-value for trend (bootstrap)	0.27			0.81			0.69			0.12			0.02		
Overall effect (Ψ, 95% CI) non-bootstrap	0.84 (−0.87, 2.55)	−0.19 (−2.70, 2.33)	1.79 (−0.55, 4.13)	1.38 (−0.61, 3.37)	0.87(−2.04, 3.77)	2.07 (−0.68, 4.82)	1.34 (−0.88, 3.56)	−0.89 (−4.04, 2.27)	3.59 (0.41, 6.76)	0.31 (−2.26, 2.89)	−0.75 (−4.36, 2.87)	2.00(−1.71, 5.71)	−0.38 (−3.00, 2.24)	−3.40 (−7.04, 0.24)	3.04 (−0.77, 6.85)
Child sex-effect modifier p-value for trend (non-bootstrap)	0.20			0.77			0.65			0.10			0.01		
Scaled negative effect	−3.38	−5.23	−5.42	−3.3	−4.95	−5.48	−6.12	−9.07	−5.38	−5.57	−8.92	−9.06	−6.65	−11.11	−7.97
Scaled positive effect	4.22	5.04	7.21	4.68	5.81	7.55	7.46	8.18	8.97	5.88	8.17	11.06	6.27	7.7	11.01
Pooled estimates using multiple imputed data – bootstrap model (95% CI)	0.88 (−0.86, 2.62)	−0.22 (−2.93, 2.49)	1.92 (−0.55, 4.39)	1.42 (−0.63, 3.46)	0.82 (−2.35, 3.98)	2.21(−0.71, 5.13)	1.28 (−1.04, 3.59)	−0.95 (−4.13, 2.24)	3.56 (0.39, 6.74)	0.39 (−2.04, 2.83)	−0.70 (−4.61, 3.22)	2.21 (−1.24, 5.66)	−0.26 (−2.92, 2.39)	−3.33 (−7.26, 0.60)	3.30 (−0.22, 6.82)
Pooled estimates using multiple imputed data – non-bootstrap model (95%CI)	0.88 (−0.84, 2.59)	0.22 (−2.73, 2.29)	1.92 (−0.44, 4.27)	1.42 (−0.57, 3.41)	0.82 (−2.08, 3.72)	2.21 (−0.56, 4.98)	1.28 (−0.95, 3.50)	−0.95 (−4.11, 2.22)	3.56 (0.36, 6.77)	0.39 (−2.19, 2.98)	−0.70 (−4.34, 2.94)	2.21 (−1.51, 5.94)	−0.26 (−2.90, 2.37)	−3.33 (−7.00, 0.35)	3.30 (−0.53, 7.13)
Chemical biomarker weights															
Pb	0.16	0.15	0.08	0.14	0.13	0.08	0.1	0.13	0.05	0.04	0.06	−0.01	0.13	0.12	0.03
Hg	−0.07	−0.03	−0.03	−0.01	0.02	−0.02	−0.03	0	−0.05	−0.01	−0.03	0.04	−0.05	−0.07	0.03
DMA	−0.05	−0.06	−0.08	−0.09	−0.1	−0.07	0.03	0	0	−0.05	−0.03	−0.08	0.02	0.05	−0.05
DDE	−0.21	−0.14	−0.13	−0.12	−0.11	−0.04	−0.06	−0.06	−0.01	−0.14	−0.1	−0.05	−0.19	−0.11	−0.09
PBDE47	0.03	−0.02	0.06	0.01	0.01	0.03	−0.02	−0.01	0.01	0.13	−0.02	0.16	0	−0.09	0.11
PCB118	0.04	0.05	−0.01	0.01	0.08	−0.11	0	−0.1	0.06	−0.17	−0.07	−0.08	−0.11	0.04	−0.19
PCB138	0	0.05	−0.12	0.09	−0.01	−0.06	−0.14	0.22	0.02	0.16	−0.02	0.14	0.09	0	0.05
PCB153	0.04	0.1	0.07	0	0.13	0.12	−0.37	−0.24	−0.43	0.2	0.33	−0.06	0.27	0.34	0.08
PCB180	0.05	−0.12	0.12	−0.01	−0.2	0.07	0.13	0.05	0.23	−−0.14	−−0.18	0.05	−0.17	−0.21	0.03
PFHxS	0.12	0.1	0.09	0.12	0.07	0.11	0.05	0.1	0	0.05	0.09	0.02	0.06	0.06	0.04
PFOA	−0.13	0.04	−0.28	−0.18	0.09	−0.32	0.02	0	−0.12	0.02	0.1	−0.1	0.01	0.1	−0.05
PFOS	−0.14	−0.14	0	−0.16	−0.15	0	−0.06	−0.1	0.05	−0.05	−0.03	−0.02	−0.09	−0.07	−0.05
BCEP	0.04	0.01	0.13	0.05	0	0.16	−0.06	0.07	0	0.02	−0.03	0.08	0.03	0.02	0.09
BDCIPP	−0.01	−0.06	0	−0.05	−0.13	−0.03	0.03	−0.01	0.1	0.03	0.05	−0.04	0.05	0.08	−0.04
DNBP	0	0.05	−0.09	−0.02	0.02	−0.07	0.04	0.07	−0.02	−0.07	0.07	−0.22	−0.06	−0.01	−0.21
DPHP	−0.08	0	−0.13	−0.07	−0.03	−0.13	−0.08	−0.01	−0.15	−0.06	−0.01	−0.06	−0.05	−0.03	−0.05
Triclosan	0.09	0.16	−0.02	0.08	0.16	−0.04	0.08	0.11	0.05	−0.02	0.04	−0.09	0.01	0.09	−0.09
Bisphenol-A	0.15	0.11	0.06	0.11	0.08	0.08	0.04	0.04	0	0.14	−0.01	0.12	0.14	−0.04	0.15
MBP	0.09	0.02	0.05	0.03	0.07	−0.06	0.07	0.03	0.04	−0.05	−0.04	−0.07	−0.04	0	−0.07
MBzP	0.09	0.05	0.04	0.07	−0.04	0.04	0.06	0.02	0.06	0.15	0.13	0.07	0.09	0.01	0.07
MCPP	0	0.06	−0.06	0.04	0.03	−0.01	−0.06	−0.03	−0.13	0.03	0.06	−0.01	0.05	0.09	−0.03
− DEHP	−0.14	−0.09	0.01	−0.13	0	0	0.04	−0.11	0.16	−0.04	0.02	0.01	−0.03	−0.02	0.06
MEP	0.01	−0.08	0.09	0.07	−0.01	0.11	−0.02	−0.05	0.05	−0.08	−0.19	0.09	−0.09	−0.17	0.08
MiBP	−0.07	−0.11	0.05	0.02	−0.05	0.09	−0.06	−0.02	−0.05	0.01	−0.06	0.07	−0.02	−0.05	0.04
Σ DEP	0.05	0.02	0.04	0.11	0.04	0.1	0.04	0.09	−0.01	0.02	−0.06	0.08	0.04	−0.03	0.1
Σ DMP	−0.06	−0.07	0.06	−0.07	−0.05	0.03	−0.09	−0.06	−0.04	0.01	0.02	0.03	−0.01	−0.01	0.02
Butyl paraben	0.03	0.04	0.02	0.05	0.08	0	0.04	0.1	0.02	0	0.01	−0.01	0	−0.01	−0.01
Methyl paraben	−0.02	0	−0.05	−0.03	−0.09	−0.01	0.09	0.04	0.08	−0.04	0.03	−0.09	−0.05	−0.02	−0.07
Propyl paraben	−0.03	−0.08	0.03	−0.06	−0.02	−0.03	−0.08	−0.11	0	−0.07	−0.13	0.03	−0.04	−0.07	0.01

Results presented in this table are generated using quantile g-computation. Numeric values for negative biomarker contributions were multiplied by —1 for presentation purposes. We considered 0.07 (2/29 = 0.069) as a threshold for highlighting the chemical biomarker weights. The interpretation of the regression coefficients is the change in cognitive score for every quartile increase in chemical biomarker mixture. Regression estimates were adjusted for child sex (while analyzing male and females combined), caregiving environment, maternal race, education, age at delivery, parity, prenatal cotinine concentrations, maternal depression, city, and cohort. Refer Table S3., to compare joint associations using multiple imputed data for unmeasured HOME scores.

## Data Availability

Due to data user agreements, the data analyzed in this study are not publicly available. The HOME study data can be requested at: https://homestudy.research.cchmc.org/contact, and the MIREC study data can be requested at: https://www.mirec-canada.ca/en/contact-us.

## Appendix A. Supplementary data

Supplementary data to this article can be found online at https://doi.org/10.1016/j.envint.2026.110383.

## Abbreviations:

Pb: Lead
Hg: Mercury
DMA: Dimethylarsinic acid
DDE: p,p’-Dichlorodiphenyldichloroethylene
PBDE47: 2,2′4,4′-tetrabromodiphenyl ether
PCB118: 2,3′,4,4′,5-Pentachlorobiphenyl
PCB138: 2,2′,3,4,4′,5′-Hexachlorobiphenyl
PCB153: 2,2′,4,4′,5,5′-Hexachlorobiphenyl
PCB180: 2,2′,3,4,4′,5,5′-Heptachlorobiphenyl
PFHxS: Perfluorohexanesulfonate
PFOA: Perfluorooctanoate
PFOS: Perfluorooctanesulfonate
BCEP: bis(2-chloroethyl) phosphate
BDCIPP: bis(1,3-dichloro-2-propyl) phosphate
DNBP: di-n-butyl phosphate
DPHP: Diphenyl phosphate
MBP: Mono-n-butyl phthalate
MBzP: Mono-benzyl phthalate
MCPP: Mono (3-carboxypropyl) phthalate
MEHHP: Mono 2-ethyl-5-hydroxyhexyl phthalate
MEHP: Mono-2-ethylhexyl phthalate
MEOHP: Mono-2-ethyl-5-oxohexyl phthalate
MECPP: Mono-2-ethyl-5-carboxypentyl phthalate
DEHP: di(2-ethylhexyl) phthalate
MEP: Monoethyl phthalate
MiBP: Monoisobutyl phthalate
DEP: Diethylphosphate
DMP: Dimethylphosphate
TC: total cholesterol
FC: free cholesterol
TG: triglycerides
PL: phospholipids
SG: specific gravity
BASC-2: Behavioral Assessment System for Children-2
BRIEF-P: Behavior Rating Inventory of Executive Function – Preschool
BSI: Behavioral Symptom Index
EXT: Externalizing Problems Index
INT: Internalizing Problems Index
PO: Planning/organizing
WM: Working Memory
HOME score: Home Observation for Measurement of the Environment score
HOME Study: Health Outcomes and Measures of the Environment Study
MIREC: Maternal-Infant Research on Environmental Chemicals
qgcomp: quantile g-computation
CEHC: Children’s Environmental Health Center
TIDES: The Infant Development and Environment Study
PACE: Pregnancy And Childhood Epigenetics
EARTH: Environment and Reproductive Health
NBC: New Bedford Cohort
CHAMACOS: Center for the Health Assessment of Mothers and Children of Salinas
APrON: Alberta Pregnancy Outcomes and Nutrition
GRowth: Programming Research in Obesity
PROGRESS: Environment and Social Stress
ELEMENT: Early Life Exposures in Mexico to ENvironmental Toxicants
MoBa: Norwegian Mother and Child Cohort Study
DNBC: Danish National Birth Cohort
SBC: Shanghai Birth Cohort
GESTE: GEStation Thyroid and Environment
