# Supplementary material for: Joint associations between gestational environmental chemical mixtures and child behavioral outcomes
Source: Environ Int. Author manuscript; Available in PMC 2026 Aug 20. (PMC13492037; doi:10.1016/j.envint.2026.110383)
Supplement: Supplementary file 1 [file NIHMS2200263-supplement-2.pdf]

Joint associations between gestational environmental chemical mixtures and child behavioral outcomes

Jagadeesh Puvvula, Wei-Ting Hwang, Lawrence McCandless, Changchun Xie, Joseph M. Braun, Ann M. Vuong, Enrique F. Schisterman, Russell T. Shinohara, Youssef Oulhote, Linda Booij, Jean R. Séguin, Maryse F. Bouchard, Christine Till, Angelika Zidek, Michael M. Borghese, Mandy Fisher, William D. Fraser, Gina Muckle, Kimberly Yolton, Kim M. Cecil, Jillian Ashley-Martin, Tye E. Arbuckle, Bruce Lanphear, Aimin Chen

*Corresponding author: Jagadeesh Puvvula – 3600 Civic Center Blvd, Philadelphia, PA 19104 USA. Email: Jagadeesh.Puvvula@pennmedicine.upenn.edu

Contents (21 pages): 6 tables, 9 figures, and biomarker assays

Table of contents

eTable 1. Level of detection (LOD) and frequency below LOD of chemical biomarkers included in this study (n=695) 2
eTable 2. Summary of participant characteristics 4
eTable 3. Associations between single chemical biomarkers and child behavioral outcomes (n=695; Females=353, Males=342). 5
eTable 4. Joint associations between gestational exposure biomarkers and child behavioral outcomes using multiple imputed data (n=695; Females=353; Males=342) 7
eTable 5. Joint associations between maternal chemical biomarker mixture and child behavioral outcomes – Stratified by cohort 8
eTable 6. PubMed search query on June 02, 2025 9

eFigure 1. Schematic representation of study participants. 10
eFigure 2. Illustration of participants enrolled in the HOME and MIREC studies with unmeasured gestational chemical biomarkers or child behavioral assessments (n=2,299). 11
eFigure 3. Directed Acyclic Graph – Conceptualizing the association between gestational chemical biomarkers and child cognitive outcomes. 12
eFigure 4. Spearman correlation between gestational chemical biomarkers, child behavioral outcomes, and covariates measured on a continuous scale. 13
eFigure 5. Joint associations between gestational chemical biomarkers and child behavioral outcomes (n=695; Females=353, Males=342). 14
eFigure 6. Gestational chemical biomarker weights contributed to the joint associations (n=695; Females=353, Males=342). 15
eFigure 7. Single gestational chemical biomarker associations with child behavioral outcomes (HOME study n= 139 [Males=66 & Females= 73]; MIREC n= 556 [Males=276 & Females= 280]). 16
eFigure 8. Single gestational chemical biomarker associations with child behavioral symptom index using maximum study participants 17
eFigure 9. Joint association between gestational chemical biomarkers and child behavioral outcomes by study sites. 18

eMethods 1. Biomarker Measurements 19

eTable 1. Level of detection (LOD) and frequency below LOD of chemical biomarkers included in this study (n=695)

Chemical group	Sample media	Chemical biomarker	Abbreviation	MIREC		HOME	
				LOD	n(%)	LOD	n(%)
1. Metals	Blood	Lead	Pb	1	0 (0)	1.8	2 (1)
	Blood	Mercury	Hg	0.1	37 (7)	0.6	27 (19)
	Urine	Dimethylarsinic acid	DMA	0.1	0 (0)	0.05	0 (0)
2. Persistent Organic Pollutants (POPs)*	Serum	p,p'-Dichlorodiphenyldichloroethylene	DDE	0.09	0 (0)	3.98	0 (0)
	Serum	2,2'4,4'-tetrabromodiphenyl ether	PBDE47	0.03	0 (0)	0.87	0 (0)
	Serum	2,3',4,4',5-Pentachlorobiphenyl	PCB118	0.01	0 (0)	0.52	1 (1)
	Serum	2,2',3,4,4',5'-Hexachlorobiphenyl	PCB138	0.01	0 (0)	0.61	0 (0)
	Serum	2,2',4,4',5,5'-Hexachlorobiphenyl	PCB153	0.01	0 (0)	0.64	0 (0)
	Serum	2,2',3,4,4',5,5'-Heptachlorobiphenyl	PCB180	0.01	0 (0)	0.52	0 (0)
3. Perfluoroalkyl substances (PFAS)	Serum	Perfluorohexanesulfonate	PFHxS	0.3	28 (5)	0.1	1 (0)
	Serum	Perfluorooctanoate	PFOA	0.1	0 (0)	0.1	0 (0)
	Serum	Perfluorooctanesulfonate	PFOS	0.3	0 (0)	0.1	0 (0)
4. Organophosphate Esters (OPEs)	Urine	bis(2-chloroethyl) phosphate	BCEP	0.02	9 (2)	0.1	14 (10)
	Urine	bis(1,3-dichloro-2-propyl) phosphate	BDCIPP	0.05	57 (10)	0.1	6 (4)
	Urine	di-n-butyl phosphate	DNBP	0.05	182 (33)	0.1	24 (17)
	Urine	Diphenyl phosphate	DPHP	0.1	77 (14)	0.1	1 (1)
5. Phenols	Urine	Triclosan	TCS	0.3	19 (3)	0.4	7 (5)
	Urine	Bisphenol-A	BPA	0.2	76 (14)	0.4	13 (9)
6. Phthalates	Urine	Mono-n-butyl phthalate	MBP	0.2	1 (0)	0.6	0 (0)
	Urine	Mono-benzyl phthalate	MBzP	0.2	3 (1)	0.3	2 (1)

	Urine	Mono(3-carboxypropyl) phthalate	MCPP		0.2	102 (18)	0.2	4 (3)
	Urine	Mono 2-ethyl-5-hydroxyhexyl phthalate	MEHHP	Σ-DEHP	0.4	2 (0)	0.7	2 (1)
	Urine	Mono-2-ethylhexyl phthalate	MEHP	Σ-DEHP	0.2	6 (1)	1.2	29 (21)
	Urine	Mono-2-ethyl-5-oxohexyl phthalate	MEOHP	Σ-DEHP	0.2	2 (0)	0.7	1 (1)
	Urine	Mono-2-ethyl-5-carboxypentyl phthalate	MECPP	Σ-DEHP	0.3	2 (0)	0.6	0 (0)
	Urine	Monoethyl phthalate	MEP		0.5	1 (0)	0.8	0 (0)
	Urine	Monoisobutyl phthalate	MiBP		0.3	10 (2)	0.3	6 (4)
7. Organophosphate (OP) pesticides	Urine	Diethylphosphate	DEP	Σ-DEP	1	129 (23)	0.559	57 (41)
	Urine	Diethylthiophosphate	DETP	Σ-DEP	0.6	299 (54)	0.379	75 (54)
	Urine	Dimethylphosphate	DMP	Σ-DMP	1	119 (21)	0.617	54 (39)
	Urine	Dimethylthiophosphate	DMTP	Σ-DMP	0.6	105 (19)	0.162	17 (12)
8. Parabens	Urine	Butyl paraben			0.7	0 (0)	0.2	44 (32)
	Urine	Methyl paraben			0.3	0 (0)	1	0 (0)
	Urine	Propyl paraben			0.2	0 (0)	0.2	1 (1)
Cotinine	a*	Cotinine	Cotinine		0.4/ 0.005	282 (51)	0.015	45 (32)

Above chemical biomarkers were measured as ng/mL, except POPs measured as ng/g of lipid; a- Serum samples in HOME Study and plasma in MIREC Study were utilized to quantify cotinine concentrations; Σ-DEHP = molar sum of MEHHP, MEHP, MECPP and MEOHP metabolite concentrations; DEP = molar sum of DEP and DETP metabolite concentrations; DMP = molar sum of DMP and DMTP metabolite concentrations. POPs contain chemical biomarkers from three distinct lipophilic chemical classes such as DDE, PBDEs, and PCBs

eTable 2. Summary of participant characteristics

		Subjects included in this study			Subjects excluded from this study		
Variable	Units	Pooled (n=695)	HOME (n=139)	MIREC (n=556)	Pooled (n=1604)	HOME (n=250)	MIREC (n=1354)
Maternal age at delivery	Years	33.00 (29.43, 36.00)	30.13 (26.22, 33.21)	33.00 (30.00 , 37.00)	32.00 (28.00, 36.00)<br>NA: 7 (0.44)	29.29 (24.67, 32.83)<br>NA: 0	32.00 (29.00, 36.00)<br>NA: 7 (0.52)
Home Observation for Measurement of the Environment (HOME) score	NA	46.70 (43.00, 49.05)	41.00 (39.00, 43.00)	48.00 (45.58, 50.00)	42.00 (39.40, 45.00)<br>NA: 1226 (76.43)	39.70 (39.00, 42.75)<br>NA: 0	47.00 (44.00, 50.00)<br>NA: 1226 (90.55)
Maternal depression	NA	5 (3, 9)	8 (5, 12)	5 (2, 8)	6 (3, 10)<br>NA: 1058 (65.96)	9 (5, 14)<br>NA: 14 (5.60)	5 (3, 7)<br>NA: 1044 (77.10)
Child sex	NA	Female: 353 (50.79)<br>Male: 342 (49.21)	Female: 73 (52.52)<br>Male: 66 (47.48)	Female: 280 (50.36)<br>Male: 276 (49.64)	Female: 716 (44.64)<br>Male: 811 (50.56)<br>NA: 77 (4.8)	Female: 135 (54.00)<br>Male: 115 (46.00)<br>NA: 0	Female: 581 (42.91)<br>Male: 696 (51.40)<br>NA: 77 (5.68)
Race	NA	White: 596 (85.76)<br>Other: 99 (14.24)	White: 100 (71.94)<br>Other: 39 (28.06)	White: 496 (89.21)<br>Other: 60 (10.79)	White: 1260 (78.55)<br>Other: 334 (20.82)<br>NA: 10 (0.62)	White: 133 (53.20)<br>Other: 107 (42.80)<br>NA: 10 (4.00)	White: 1127 (83.23)<br>Other: 227 (16.77)<br>NA: 0
Parity	NA	0: 307 (44.17)<br>1: 279 (40.14)<br>2: 84 (12.09)<br>>3: 25 (3.59)	0: 62 (44.60)<br>1: 44 (31.65)<br>2: 26 (18.71)<br>>3: 7 (5.04)	0: 245 (44.06)<br>1: 235 (42.27)<br>2: 58 (10.43)<br>>3: 18 (3.24)	0: 704 (43.89)<br>1: 619 (38.59)<br>2: 201 (12.53)<br>>3: 73 (4.54)<br>NA: 7 (0.44)	0: 107 (42.80)<br>1: 85 (34.00)<br>2: 36 (14.40)<br>>3: 22 (8.80)<br>NA: 0	0: 597 (44.09)<br>1: 534 (39.44)<br>2: 165 (12.19)<br>>3: 51 (3.76)<br>NA: 7 (0.52)
Education	NA	L1: 92 (13.24)<br>L2: 417 (60.00)<br>L3: 186 (26.76)	L1: 57 (41.01)<br>L2: 51 (36.69)<br>L3: 31 (22.30)	L1: 35 (6.29)<br>L2: 366 (65.83)<br>L3: 155 (27.88)	L1: 260 (16.21)<br>L2: 948 (59.10)<br>L3: 378 (23.57)<br>NA: 18 (1.12)	L1: 128 (51.20)<br>L2: 63 (25.20)<br>L3: 48 (19.20)<br>NA: 11 (4.40)	L1: 132 (9.75)<br>L2: 885 (65.36)<br>L3: 330 (24.37)<br>NA: 7 (0.52)
Cotinine	ng/mL	0.01 (<LOD, 0.03)	0.02 (<LOD, 0.09)	0.01 (<LOD, 0.03)	0.01 (<LOD, 0.06)<br>NA: 99 (6.17)	0.05 (<LOD, 0.61)<br>NA: 8 (3.20)	0.01 (<LOD, 0.04)<br>NA: 91 (6.72)

The above table contains median and interquartile ranges for continuous variables, count, and percentages for categorical variables. LOD: Level of detection; Education: L-1=less than or equal to high school education, L-2=greater than high school and less than a postgraduate education, and L-3= postgraduate or more; NA values in this table represent unmeasured observations.

Table 3. Associations between single chemical biomarkers and child behavioral outcomes (n=695, Females=353, Males=342).

	BASC									BRIEF					
	BSI			EXT			INT			PO			WM		
	All	Females	Males	All	Females	Males	All	Females	Males	All	Females	Males	All	Females	Males
Pb	1.01 (0.23, 1.78)	1.05 (-0.13, 2.24)	0.91 (-0.13, 1.95)	1.13 (0.23, 2.02)	1.21 (-0.14, 2.57)	1.15 (-0.07, 2.37)	1.09 (0.08, 2.10)	1.11 (-0.41, 2.62)	1.02 (-0.36, 2.41)	0.21 (-0.96, 1.38)	-0.12 (-1.84, 1.60)	0.21 (-1.44, 1.87)	0.98 (-0.21, 2.16)	1 (-0.75, 2.76)	0.56 (-1.13, 2.24)
Hg	-0.11 (-0.48, 0.26)	0.04 (-0.47, 0.54)	-0.21 (-0.75, 0.32)	-0.11 (-0.53, 0.31)	0.13 (-0.45, 0.71)	-0.28 (-0.91, 0.34)	-0.02 (-0.49, 0.46)	0.12 (-0.52, 0.77)	-0.17 (-0.88, 0.54)	0.16 (-0.39, 0.71)	-0.02 (-0.76, 0.71)	0.47 (-0.37, 1.32)	-0.01 (-0.57, 0.55)	-0.11 (-0.86, 0.64)	0.17 (-0.70, 1.03)
DMA	0.16 (-0.33, 0.65)	0.11 (-0.62, 0.83)	0.16 (-0.50, 0.82)	0.15 (-0.41, 0.72)	0.13 (-0.69, 0.96)	0.1 (-0.67, 0.88)	0.35 (-0.29, 0.98)	0.19 (-0.74, 1.12)	0.4 (-0.47, 1.28)	-0.35 (-1.08, 0.39)	-0.23 (-1.28, 0.82)	-0.47 (-1.52, 0.57)	0.24 (-0.51, 0.98)	0.57 (-0.50, 1.64)	-0.05 (-1.11, 1.02)
DDE	-0.14 (-0.72, 0.43)	-0.15 (-1.00, 0.71)	-0.08 (-0.86, 0.70)	0.09 (-0.58, 0.75)	-0.03 (-1.01, 0.95)	0.28 (-0.64, 1.19)	-0.09 (-0.84, 0.65)	-0.58 (-1.68, 0.51)	0.39 (-0.64, 1.42)	-0.14 (-1.01, 0.72)	-0.51 (-1.75, 0.72)	0.23 (-1.00, 1.47)	-0.75 (-1.63, 0.13)	-1.59 (-2.85, -0.34)	0.09 (-1.17, 1.35)
PBDE47	0.21 (-0.16, 0.58)	-0.09 (-0.65, 0.46)	0.29 (-0.21, 0.79)	0.21 (-0.21, 0.64)	0.03 (-0.61, 0.66)	0.18 (-0.41, 0.76)	0.18 (-0.29, 0.66)	-0.19 (-0.90, 0.53)	0.38 (-0.28, 1.05)	0.62 (0.07, 1.17)	-0.1 (-0.91, 0.71)	1.17 (0.39, 1.96)	-0.02 (-0.58, 0.55)	-0.97 (-1.78, -0.15)	0.75 (-0.06, 1.55)
PCB118	0 (-0.63, 0.63)	0.33 (-0.61, 1.28)	-0.28 (-1.14, 0.58)	-0.02 (-0.74, 0.71)	0.5 (-0.59, 1.58)	-0.53 (-1.54, 0.47)	-0.28 (-1.09, 0.54)	-0.81 (-2.02, 0.40)	0.36 (-0.77, 1.50)	0.25 (-0.69, 1.19)	0.12 (-1.26, 1.49)	0.49 (-0.87, 1.85)	-0.08 (-1.04, 0.88)	0.31 (-1.09, 1.71)	-0.44 (-1.83, 0.94)
PCB138	0.11 (-0.50, 0.72)	0.11 (-0.76, 0.98)	0.13 (-0.75, 1.01)	0.4 (-0.31, 1.10)	0.42 (-0.57, 1.42)	0.32 (-0.72, 1.35)	-0.33 (-1.12, 0.47)	-0.97 (-2.08, 0.14)	0.55 (-0.62, 1.72)	0.42 (-0.50, 1.34)	-0.11 (-1.37, 1.15)	1.09 (-0.30, 2.48)	-0.03 (-0.97, 0.90)	-0.48 (-1.77, 0.81)	0.4 (-1.03, 1.83)
PCB153	0 (-0.62, 0.62)	-0.15 (-1.04, 0.74)	0.33 (-0.56, 1.22)	0.3 (-0.42, 1.01)	0.19 (-0.82, 1.21)	0.52 (-0.52, 1.56)	-0.46 (-1.26, 0.35)	-1.08 (-2.22, 0.05)	0.45 (-0.73, 1.62)	0.11 (-0.82, 1.04)	-0.48 (-1.77, 0.80)	0.9 (-0.50, 2.30)	-0.19 (-1.14, 0.76)	-0.76 (-2.08, 0.55)	0.46 (-0.98, 1.89)
PCB180	0.06 (-0.51, 0.64)	-0.01 (-0.82, 0.80)	0.41 (-0.41, 1.24)	0.28 (-0.38, 0.94)	0.25 (-0.68, 1.17)	0.57 (-0.40, 1.53)	-0.22 (-0.96, 0.52)	-0.53 (-1.57, 0.50)	0.43 (-0.66, 1.52)	-0.02 (-0.88, 0.83)	-0.58 (-1.75, 0.59)	0.8 (-0.51, 2.10)	-0.19 (-1.06, 0.68)	-0.75 (-1.94, 0.45)	0.5 (-0.84, 1.83)
PFHxS	0.27 (-0.20, 0.75)	0.42 (-0.28, 1.12)	0.14 (-0.52, 0.79)	0.26 (-0.29, 0.80)	0.38 (-0.42, 1.18)	0.18 (-0.59, 0.94)	0.49 (-0.12, 1.11)	0.61 (-0.28, 1.51)	0.38 (-0.49, 1.25)	0.55 (-0.16, 1.26)	1.3 (0.29, 2.31)	-0.16 (-1.20, 0.88)	0.33 (-0.39, 1.06)	0.57 (-0.47, 1.60)	0.16 (-0.90, 1.22)
PFOA	-0.88 (-1.55, -0.20)	-0.3 (-1.33, 0.72)	-1.41 (-2.32, -0.51)	-1.01 (-1.79, -0.23)	-0.16 (-1.33, 1.01)	-1.75 (-2.81, -0.69)	-0.24 (-1.12, 0.65)	-0.27 (-1.58, 1.04)	-0.39 (-1.61, 0.83)	-0.79 (-1.81, 0.23)	0.11 (-1.37, 1.60)	-1.77 (-3.21, -0.33)	-0.87 (-1.91, 0.16)	-0.44 (-1.95, 1.08)	-1.32 (-2.79, 0.16)
PFOS	-0.4 (-1.06, 0.25)	-0.25 (-1.22, 0.72)	-0.41 (-1.31, 0.50)	-0.44 (-1.20, 0.32)	-0.03 (-1.14, 1.09)	-0.66 (-1.72, 0.41)	-0.18 (-1.04, 0.67)	-0.3 (-1.54, 0.95)	-0.03 (-1.23, 1.17)	0.08 (-0.91, 1.06)	0.18 (-1.23, 1.59)	-0.15 (-1.58, 1.29)	-0.45 (-1.45, 0.55)	-0.84 (-2.28, 0.59)	-0.07 (-1.53, 1.39)
BCEP	0.06 (-0.28, 0.39)	-0.42 (-0.90, 0.06)	0.56 (0.09, 1.04)	0.13 (-0.26, 0.52)	-0.41 (-0.96, 0.14)	0.71 (0.15, 1.26)	-0.22 (-0.66, 0.21)	-0.74 (-1.35, -0.12)	0.23 (-0.40, 0.87)	0.06 (-0.45, 0.57)	-0.27 (-0.97, 0.43)	0.36 (-0.40, 1.12)	0.28 (-0.23, 0.80)	-0.17 (-0.89, 0.54)	0.8 (0.02, 1.57)
BDCIPP	-0.05 (-0.40, 0.31)	-0.37 (-0.89, 0.16)	0.22 (-0.27, 0.70)	0.06 (-0.35, 0.47)	-0.35 (-0.95, 0.26)	0.35 (-0.22, 0.92)	-0.14 (-0.60, 0.32)	-0.66 (-1.34, 0.01)	0.32 (-0.32, 0.96)	0.26 (-0.28, 0.79)	0.54 (-0.23, 1.30)	0.07 (-0.69, 0.84)	0.19 (-0.35, 0.73)	0.4 (-0.38, 1.19)	0.08 (-0.70, 0.87)
DNBP	0.24 (-0.21, 0.70)	0.3 (-0.32, 0.92)	0.1 (-0.58, 0.78)	0.28 (-0.25, 0.80)	0.2 (-0.50, 0.91)	0.27 (-0.52, 1.06)	0.38 (-0.20, 0.97)	0.4 (-0.39, 1.19)	0.36 (-0.54, 1.25)	-0.21 (-0.89, 0.47)	0.26 (-0.63, 1.16)	-1.05 (-2.11, 0.02)	-0.21 (-0.90, 0.48)	0.09 (-0.83, 1.00)	-0.73 (-1.82, 0.36)

DPHP	-0.12 (-0.48, 0.24)	-0.1 (-0.63, 0.42)	-0.2 (-0.70, 0.29)	-0.08 (-0.50, 0.33)	-0.15 (-0.75, 0.45)	-0.04 (-0.63, 0.54)	-0.19 (-0.65, 0.28)	-0.35 (-1.02, 0.32)	-0.15 (-0.81, 0.51)	-0.13 (-0.67, 0.41)	-0.14 (-0.90, 0.62)	-0.14 (-0.93, 0.64)	-0.17 (-0.72, 0.38)	-0.6 (-1.38, 0.17)	0.24 (-0.56, 1.05)
Triclosan	0.09 (-0.08, 0.25)	0.14 (-0.09, 0.38)	0.04 (-0.18, 0.27)	0.07 (-0.11, 0.26)	0.2 (-0.07, 0.46)	-0.03 (-0.29, 0.23)	0.13 (-0.08, 0.34)	0.09 (-0.21, 0.39)	0.18 (-0.12, 0.47)	-0.08 (-0.33, 0.16)	-0.02 (-0.36, 0.32)	-0.16 (-0.51, 0.20)	-0.01 (-0.25, 0.24)	0.07 (-0.28, 0.42)	-0.1 (-0.46, 0.26)
Bisphenol-A	0.54 (0.19, 0.90)	0.54 (0.05, 1.03)	0.59 (0.06, 1.13)	0.45 (0.04, 0.87)	0.39 (-0.17, 0.95)	0.65 (0.02, 1.29)	0.57 (0.10, 1.03)	0.62 (-0.01, 1.24)	0.51 (-0.20, 1.23)	0.42 (-0.12, 0.95)	-0.1 (-0.81, 0.61)	1.03 (0.18, 1.88)	0.39 (-0.16, 0.93)	-0.42 (-1.15, 0.30)	1.32 (0.46, 2.18)
MBP	0.17 (-0.24, 0.59)	-0.17 (-0.77, 0.43)	0.45 (-0.12, 1.03)	0.1 (-0.38, 0.58)	0.04 (-0.64, 0.72)	0.11 (-0.56, 0.79)	0.15 (-0.38, 0.69)	-0.14 (-0.90, 0.63)	0.28 (-0.48, 1.04)	0.21 (-0.41, 0.83)	-0.11 (-0.98, 0.76)	0.52 (-0.39, 1.43)	-0.01 (-0.64, 0.62)	-0.65 (-1.54, 0.23)	0.65 (-0.27, 1.58)
MBzP	0.28 (-0.09, 0.65)	-0.22 (-0.76, 0.31)	0.54 (0.00, 1.07)	0.27 (-0.16, 0.70)	-0.22 (-0.82, 0.39)	0.52 (-0.10, 1.15)	0.36 (-0.12, 0.85)	-0.29 (-0.97, 0.39)	0.79 (0.08, 1.49)	0.63 (0.08, 1.19)	0.38 (-0.39, 1.15)	0.71 (-0.13, 1.56)	0.29 (-0.27, 0.86)	-0.49 (-1.28, 0.30)	0.95 (0.09, 1.80)
MCPP	-0.01 (-0.33, 0.31)	-0.01 (-0.52, 0.50)	-0.01 (-0.42, 0.40)	0.15 (-0.21, 0.52)	0.14 (-0.44, 0.72)	0.2 (-0.28, 0.68)	-0.18 (-0.59, 0.23)	-0.46 (-1.11, 0.18)	-0.01 (-0.55, 0.53)	0.18 (-0.29, 0.66)	0.19 (-0.54, 0.93)	0.2 (-0.45, 0.85)	0.19 (-0.30, 0.67)	0.02 (-0.74, 0.77)	0.29 (-0.37, 0.95)
Σ DEHP	-0.19 (-0.59, 0.20)	-0.27 (-0.87, 0.33)	-0.18 (-0.71, 0.34)	-0.12 (-0.58, 0.33)	0.07 (-0.62, 0.75)	-0.29 (-0.91, 0.33)	-0.04 (-0.55, 0.48)	-0.79 (-1.55, -0.03)	0.46 (-0.24, 1.15)	-0.1 (-0.69, 0.49)	0.26 (-0.61, 1.13)	-0.54 (-1.37, 0.30)	-0.08 (-0.68, 0.53)	-0.06 (-0.95, 0.83)	-0.21 (-1.06, 0.65)
MEP	-0.06 (-0.32, 0.21)	-0.34 (-0.72, 0.04)	0.19 (-0.17, 0.56)	0.1 (-0.21, 0.40)	-0.07 (-0.50, 0.36)	0.23 (-0.20, 0.66)	-0.18 (-0.52, 0.16)	-0.56 (-1.04, -0.08)	0.2 (-0.29, 0.68)	-0.27 (-0.66, 0.13)	-0.8 (-1.34, -0.25)	0.2 (-0.38, 0.78)	-0.32 (-0.72, 0.08)	-0.91 (-1.46, -0.36)	0.28 (-0.31, 0.87)
MiBP	0.12 (-0.32, 0.56)	-0.45 (-1.09, 0.18)	0.74 (0.14, 1.35)	0.27 (-0.24, 0.77)	-0.17 (-0.90, 0.55)	0.77 (0.06, 1.48)	-0.12 (-0.69, 0.44)	-0.61 (-1.42, 0.21)	0.34 (-0.47, 1.15)	0.23 (-0.43, 0.88)	-0.63 (-1.55, 0.29)	1 (0.04, 1.97)	0.24 (-0.43, 0.91)	-0.57 (-1.51, 0.37)	1.05 (0.06, 2.03)
Σ DEP	0.03 (-0.34, 0.40)	-0.01 (-0.52, 0.49)	0.1 (-0.47, 0.67)	0.09 (-0.34, 0.52)	0.04 (-0.53, 0.61)	0.22 (-0.44, 0.89)	0.03 (-0.45, 0.52)	0.38 (-0.26, 1.02)	-0.57 (-1.32, 0.18)	-0.05 (-0.61, 0.51)	-0.48 (-1.20, 0.25)	0.58 (-0.32, 1.48)	0.13 (-0.44, 0.70)	-0.31 (-1.05, 0.44)	0.78 (-0.14, 1.69)
Σ DMP	-0.07 (-0.37, 0.23)	-0.17 (-0.62, 0.27)	0.04 (-0.38, 0.45)	-0.06 (-0.41, 0.28)	-0.09 (-0.59, 0.42)	-0.01 (-0.50, 0.47)	-0.15 (-0.54, 0.24)	-0.09 (-0.66, 0.48)	-0.21 (-0.76, 0.34)	0.11 (-0.34, 0.56)	0.13 (-0.51, 0.77)	0.11 (-0.55, 0.77)	0.15 (-0.32, 0.61)	0.2 (-0.46, 0.85)	0.09 (-0.58, 0.76)
Butyl paraben	-0.01 (-0.15, 0.14)	0.01 (-0.20, 0.21)	0 (-0.20, 0.21)	0.02 (-0.14, 0.19)	0.09 (-0.15, 0.33)	-0.05 (-0.29, 0.19)	0.07 (-0.12, 0.26)	0.13 (-0.14, 0.39)	0.05 (-0.23, 0.32)	-0.11 (-0.33, 0.11)	-0.14 (-0.44, 0.17)	-0.06 (-0.39, 0.26)	-0.14 (-0.36, 0.08)	-0.23 (-0.54, 0.08)	-0.01 (-0.34, 0.32)
Methyl paraben	-0.04 (-0.27, 0.19)	-0.12 (-0.45, 0.22)	0.1 (-0.23, 0.43)	-0.01 (-0.27, 0.26)	-0.02 (-0.39, 0.36)	0.04 (-0.35, 0.43)	0.07 (-0.23, 0.37)	-0.2 (-0.62, 0.23)	0.39 (-0.05, 0.82)	-0.31 (-0.65, 0.04)	-0.42 (-0.90, 0.06)	-0.17 (-0.69, 0.35)	-0.34 (-0.70, 0.01)	-0.53 (-1.01, -0.04)	-0.08 (-0.62, 0.45)
Propyl paraben	-0.01 (-0.20, 0.18)	-0.08 (-0.35, 0.20)	0.13 (-0.14, 0.39)	-0.02 (-0.23, 0.20)	-0.04 (-0.35, 0.27)	0.07 (-0.24, 0.38)	0.04 (-0.20, 0.29)	-0.17 (-0.52, 0.18)	0.29 (-0.06, 0.64)	-0.26 (-0.54, 0.02)	-0.49 (-0.88, -0.09)	0.01 (-0.41, 0.43)	-0.21 (-0.49, 0.08)	-0.46 (-0.86, -0.06)	0.1 (-0.32, 0.53)

Results presented in this table are generated using multiple linear regression. Regression estimates were adjusted for child sex (while analyzing male and females combined), caregiving environment, maternal race, education, age at delivery, parity, prenatal cotinine concentrations, maternal depression, city, and cohort.

eTable 4. Joint associations between gestational exposure biomarkers and child behavioral outcomes using multiple imputed data (n=695; Females=353; Males=342)

	Pooled estimates using multiple imputed data – bootstrap model (95%CI)	Pooled estimates using multiple imputed data – non-bootstrap model (95%CI)
All		
BASC-BSI	0.88 (-0.86, 2.62)	0.88 (-0.84, 2.59)
BASC-EXT	1.42 (-0.63, 3.46)	1.42 (-0.57, 3.41)
BASC-INT	1.28 (-1.04, 3.59)	1.28 (-0.95, 3.50)
BRIEF-PO	0.39 (-2.04, 2.83)	0.39 (-2.19, 2.98)
BRIEF-WM	-0.26 (-2.92, 2.39)	-0.26 (-2.90, 2.37)
Females		
BASC-BSI	-0.22 (-2.93, 2.49)	-0.22 (-2.73, 2.29)
BASC-EXT	0.82 (-2.35, 3.98)	0.82 (-2.08, 3.72)
BASC-INT	-0.95 (-4.13, 2.24)	-0.95 (-4.11, 2.22)
BRIEF-PO	-0.70 (-4.61, 3.22)	-0.70 (-4.34, 2.94)
BRIEF-WM	-3.33 (-7.26, 0.60)	-3.33 (-7.00, 0.35)
Males		
BASC-BSI	1.92 (-0.55, 4.39)	1.92 (-0.44, 4.27)
BASC-EXT	2.21 (-0.71, 5.13)	2.21 (-0.56, 4.98)
BASC-INT	3.56 (0.39, 6.74)	3.56 (0.36, 6.77)
BRIEF-PO	2.21 (-1.24, 5.66)	2.21 (-1.51, 5.94)
BRIEF-WM	3.30 (-0.22, 6.82)	3.30 (-0.53, 7.13)

Joint associations were assessed using quantile g-computation. Above results were generated using 15 multiple imputed datasets to assess potential uncertainty due to the 100 study participants with unmeasured HOME scored from the MIREC study. These results were presented complementing our findings from Table 2 that contains joint associations using HOME scores imputed using a single imputation method.

eTable 5. Joint associations between maternal chemical biomarker mixture and child behavioral outcomes – Stratified by cohort

	HOME (n=139)					MIREC (n=556)				
	BASC-BSI	BASC-EXT	BASC-INT	BRIEF-PO	BRIEF-WM	BASC-BSI	BASC-EXT	BASC-INT	BRIEF-PO	BRIEF-WM
Overall bootstrap effect (Ψ, 95% CI)	5.00 (-1.62, 11.63)	7.13 (-0.15, 14.41)	-0.25 (-9.07, 8.58)	-1.01 (-9.22, 7.21)	2.59 (-6.02, 11.20)	-0.06 (-1.65, 1.52)	0.66 (-1.32, 2.64)	0.55 (-1.61, 2.71)	0.06 (-2.34, 2.45)	-0.71 (-3.20, 1.77)
Overall effect (Ψ, 95% CI) non-bootstrap	5.00 (-0.86, 10.87)	7.13 (1.10, 13.16)	-0.25 (-7.66, 7.17)	-1.01 (-8.90, 6.89)	2.59 (-5.34, 10.52)	-0.06 (-1.68, 1.56)	0.66 (-1.31, 2.63)	0.55 (-1.61, 2.72)	0.06 (-2.53, 2.64)	-0.71 (-3.34, 1.91)
Scaled negative effect	-7.57	-7.11	-15.65	-14.27	-11.24	-4.72	-5.05	-5.06	-8.26	-8.28
Scaled positive effect	12.57	14.24	15.4	13.27	13.83	4.66	5.71	5.62	8.32	7.56
Pb	0.09	0.04	0.12	-0.01	0.07	0.12	0.11	0.06	0.03	0.1
Hg	0.06	0.05	0.05	0.02	-0.05	-0.08	-0.04	-0.12	0.03	-0.02
DMA	-0.04	-0.03	-0.08	-0.06	-0.06	0	-0.03	0.08	-0.03	0.04
DDE	-0.09	-0.03	-0.08	-0.02	-0.05	-0.09	-0.03	0.03	-0.06	-0.11
PBDE47	0.13	0.13	0.03	0.14	0.06	-0.06	-0.08	-0.06	0.05	-0.02
PCB118	0.03	0.01	0.08	0.08	0.03	-0.11	-0.09	-0.1	-0.17	-0.18
PCB138	-0.09	-0.15	-0.18	-0.21	-0.06	0	-0.07	-0.02	0.09	0.03
PCB153	0.06	0.13	0.06	0.3	0.11	0.28	0.27	-0.1	0.26	0.36
PCB180	0.01	0.01	0.1	-0.09	0	-0.11	-0.12	0.09	-0.19	-0.22
PFHxS	0.03	0.02	0.01	-0.04	-0.11	0.11	0.1	0.05	0.1	0.12
PFOA	-0.29	-0.38	-0.11	-0.12	-0.18	-0.12	-0.11	-0.05	-0.06	-0.03
PFOS	0.11	0.11	0.09	-0.02	0.11	-0.02	-0.04	0.04	-0.04	-0.05
BCEP	0.08	0.05	0.03	0.06	0.05	0.04	0.06	-0.05	-0.01	0.02
BDCIPP	-0.18	-0.09	-0.15	-0.12	-0.12	0.02	-0.03	0.07	0.12	0.1
DNBP	0.03	0.03	0.03	0.04	0.05	-0.03	-0.03	0.03	-0.09	-0.12
DPHP	0.01	0	0	0.04	0.03	-0.06	-0.04	-0.03	-0.09	-0.05
Triclosan	0.08	0.11	0.07	0	0.08	0.05	0.02	0.04	-0.03	-0.01
Bisphenol-A	0.08	0.08	0.08	0.17	0.16	0.15	0.1	0.07	0.08	0.1
MBP	-0.01	-0.02	-0.08	0.07	0.01	-0.02	-0.02	0	-0.09	-0.07
MBzP	0.01	-0.04	0.13	-0.01	-0.01	0.12	0.11	0.06	0.12	0.07
MCPP	-0.01	-0.02	-0.06	-0.13	-0.11	-0.07	-0.01	-0.12	0.04	0
Σ DEHP	-0.05	-0.01	-0.11	-0.01	0.05	-0.02	-0.05	0.16	-0.02	0.01
MEP	0.04	0.03	0.01	0.04	0.01	0	0.05	-0.03	-0.03	-0.03
MiBP	0.06	0.09	0.11	0.03	-0.05	-0.06	0	-0.09	0.05	0.01
Σ Di-ethyl organophosphate	0.01	0.02	0	0.02	0.09	0.05	0.08	0.01	0.01	0.03
Σ Di-methyl organophosphate	-0.01	0	-0.02	-0.04	-0.1	-0.04	-0.04	-0.1	-0.01	0
Butyl paraben	0.01	0.06	-0.01	0	0.07	-0.02	-0.04	0	-0.02	-0.03
Methyl paraben	-0.24	-0.22	-0.11	-0.03	-0.1	0.08	0.1	0.19	0.03	-0.03
Propyl paraben	0.09	0.03	-0.01	-0.09	0.03	-0.08	-0.12	-0.11	-0.07	-0.05

Results presented here were generated using the quantile g-computation regression. The interpretation of the regression coefficients is the change in cognitive score for every quartile increase in chemical biomarker mixture. Regression estimates were adjusted for child sex (while analyzing male and females combined), caregiving environment, maternal race, education, age at delivery, parity, maternal depression, gestational cotinine concentrations.

eTable 6. PubMed search query on June 02, 2025

(gestational OR pregnancy OR prenatal) AND (“metal*” OR "polybrominated diphenyl ether*" OR PBDE OR "polychlorinated biphenyl*" OR PCB OR "perfluoroalkyl substance*" OR PFAS OR "bisphenol*" OR phthalate* OR triclosan OR "organophosphate ester*" OR pesticide* OR paraben* OR phenol*) AND ("Behavior Assessment System for Children" OR "Behavior Rating Inventory of Executive Function") AND humans[MeSH Terms] AND English[Language]
Identified a total of 37 articles. A review article and a retracted article were excluded for review in this study.

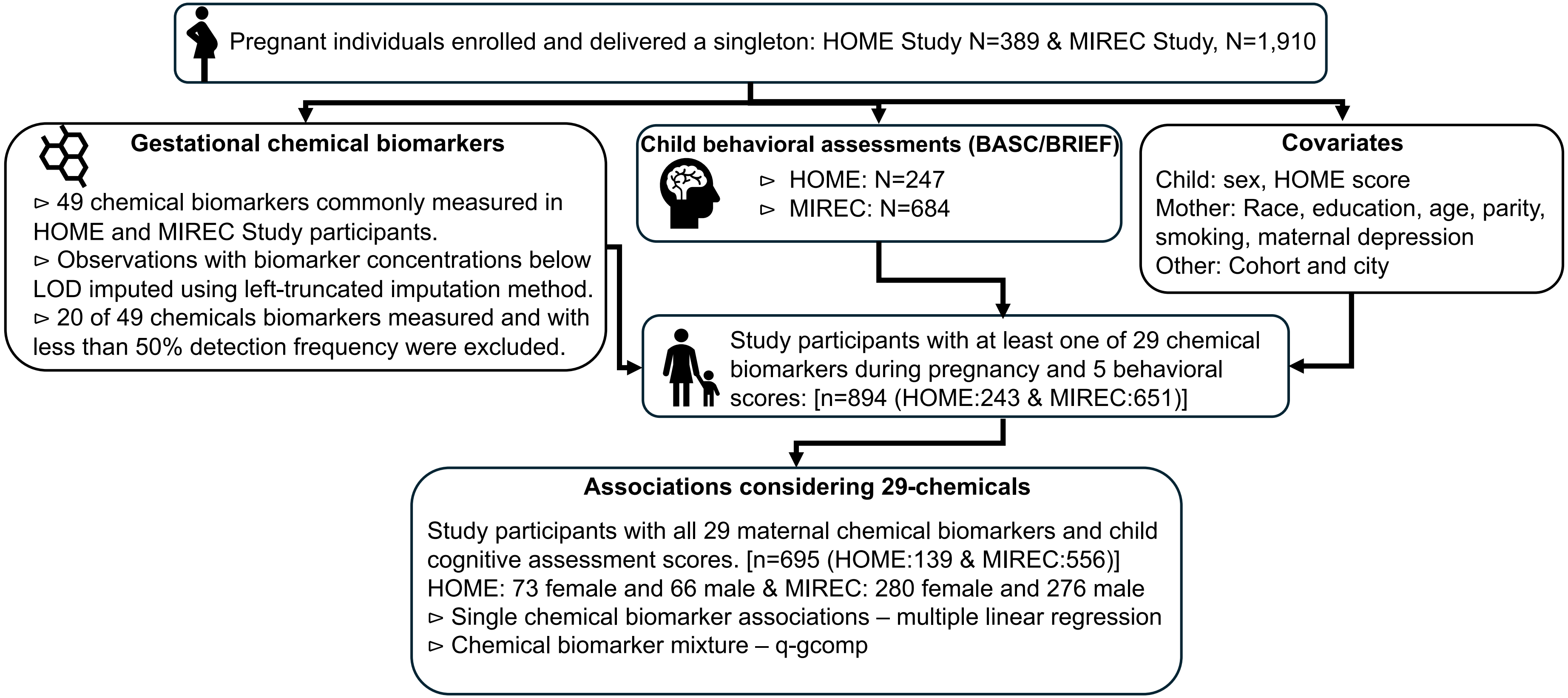

eFigure 1. Schematic representation of study participants.

In the final analytic dataset from MIREC Study, 556 participants were from five Canadian cities. Vancouver – 54; Toronto – 56; Hamilton – 75; Kingston – 104; Montreal – 132; Halifax – 135.

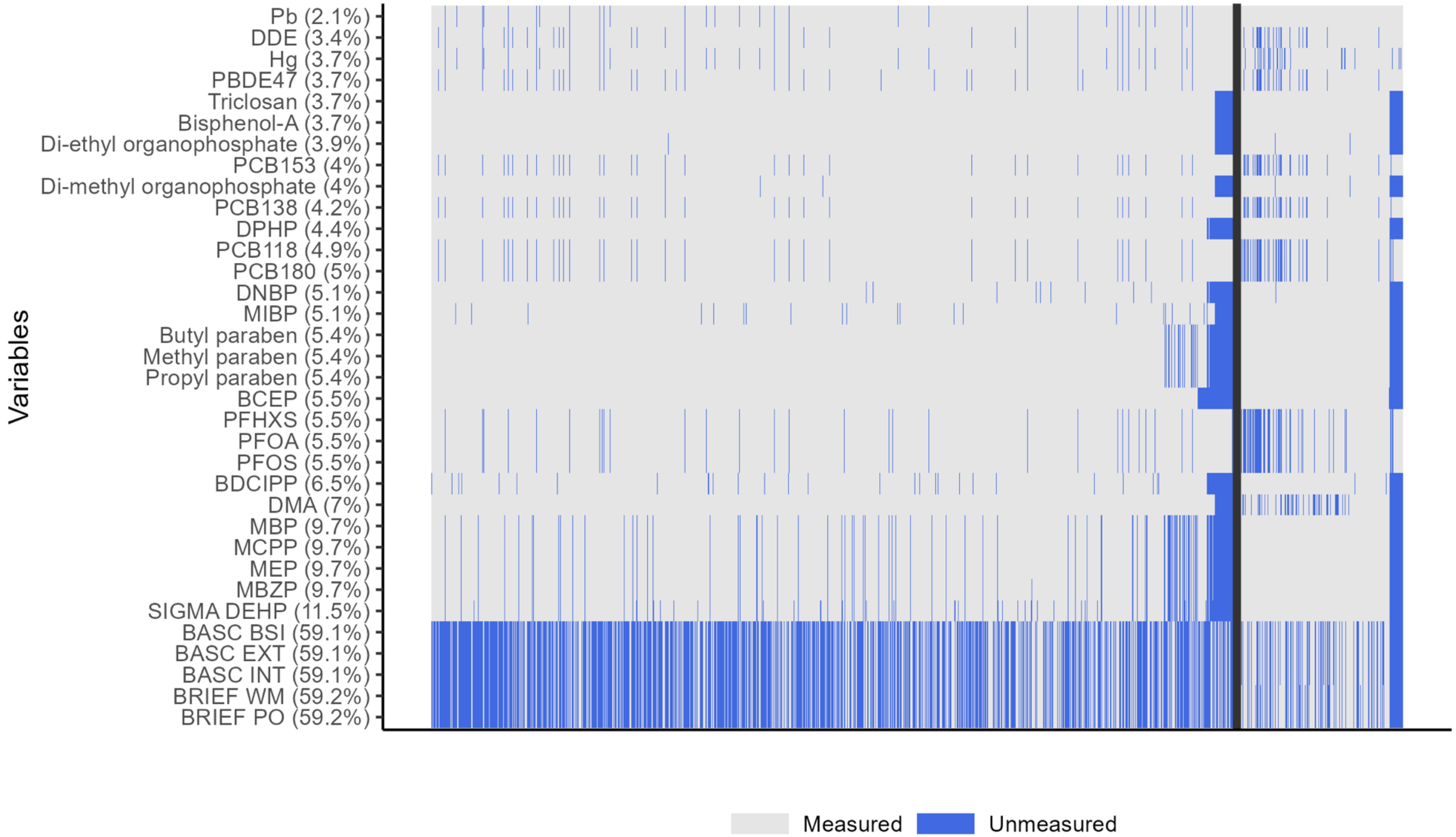

eFigure 2. Illustration of participants enrolled in the HOME and MIREC studies with unmeasured gestational chemical biomarkers or child behavioral assessments (n=2,299).

The y-axis contains chemical biomarkers and cognitive outcomes (sorted by percent of unmeasured observations), along with the percentage of unmeasured observations. The x-axis represents subjects enrolled in the HOME and MIREC studies. Each vertical blue bar highlights unmeasured observations. The vertical black line partitions the MIREC and HOME study participants.

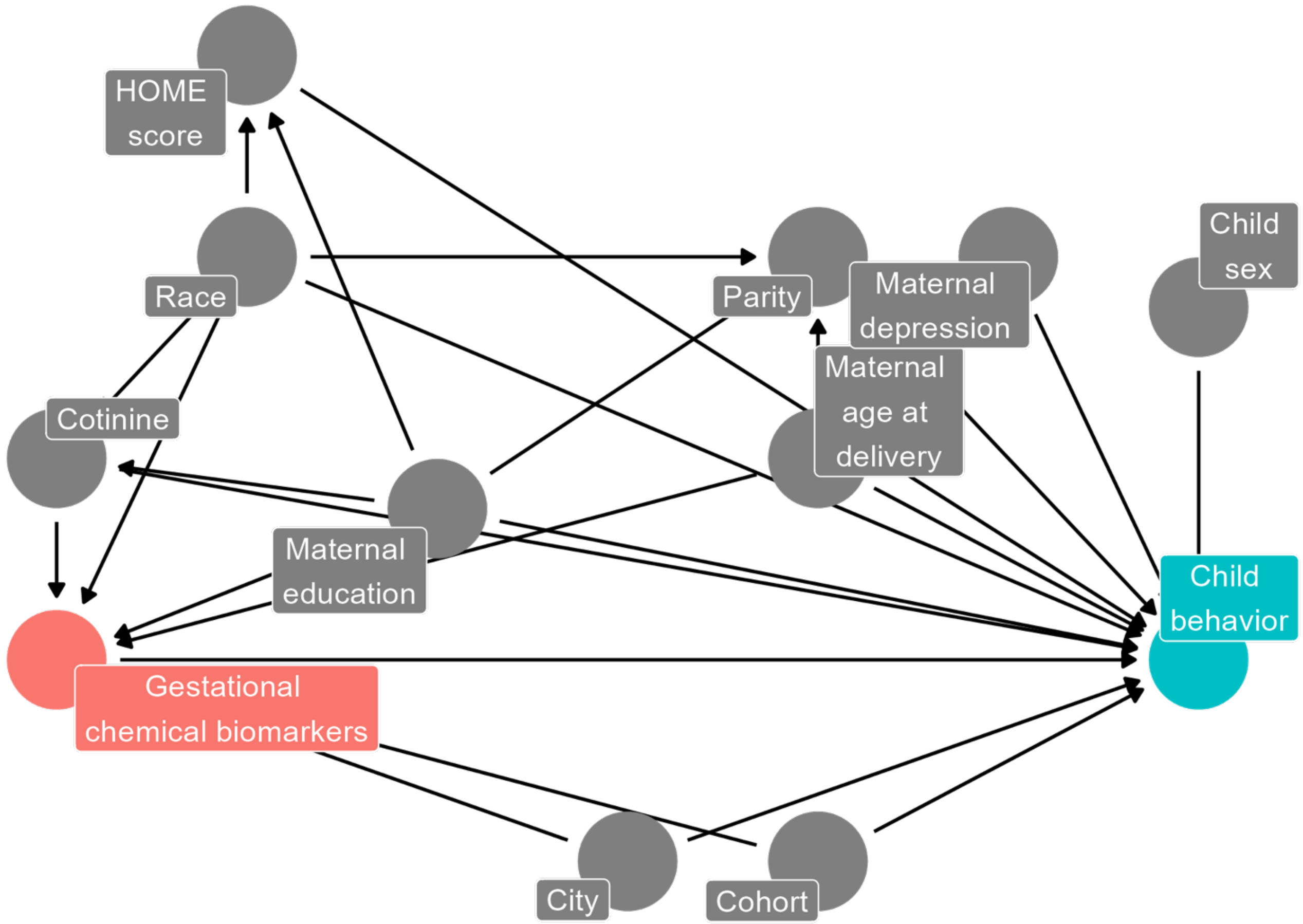

eFigure 3. Directed Acyclic Graph – Conceptualizing the association between gestational chemical biomarkers and child cognitive outcomes.

The exposure (chemical biomarker mixture) variable is color-coded in red; the outcome (child behavioral score) is coded in cyan, and potential covariates are color-coded in gray and were adjusted for the exposure-response association.

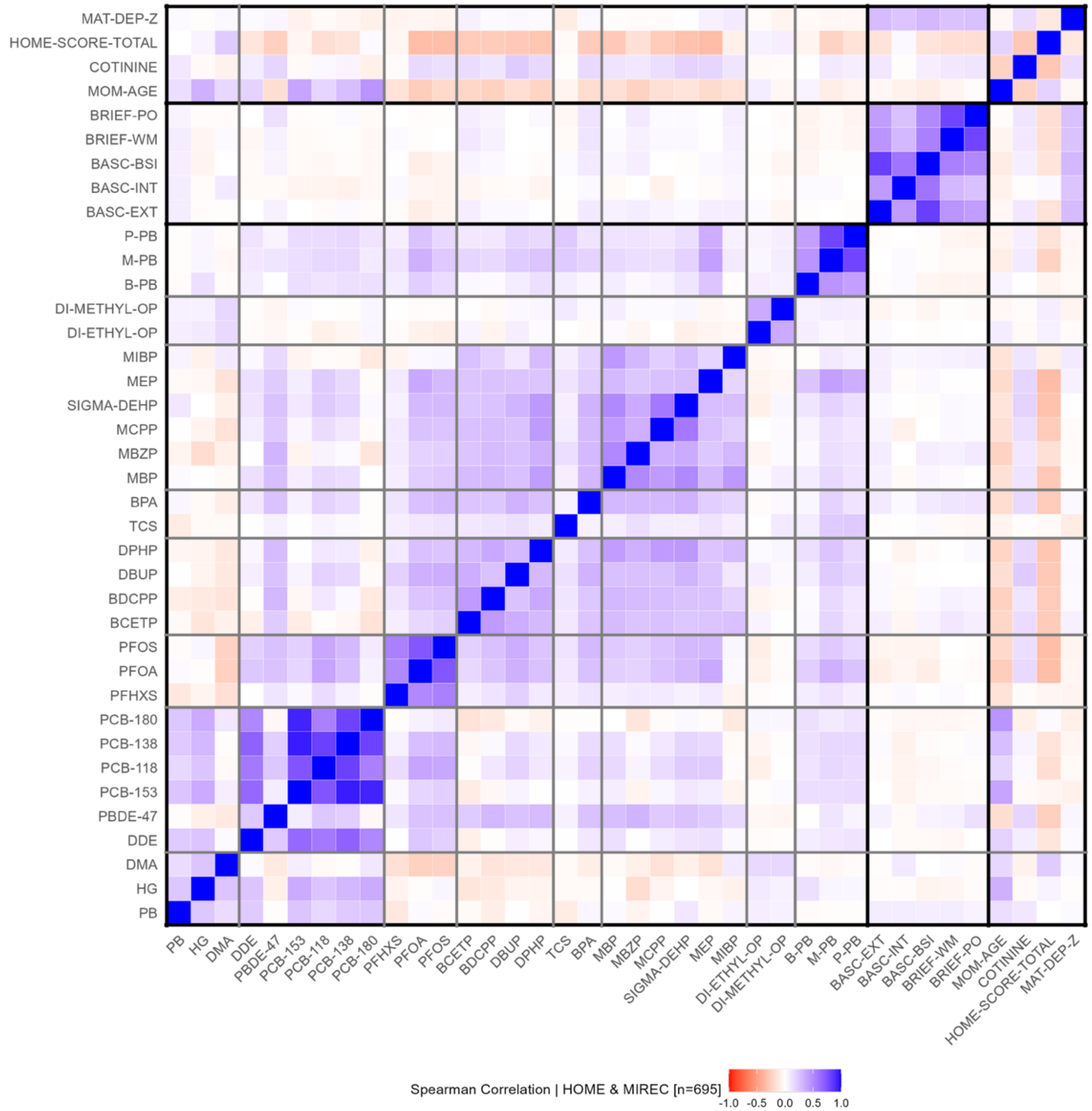

eFigure 4. Spearman correlation between gestational chemical biomarkers, child behavioral outcomes, and covariates measured on a continuous scale.

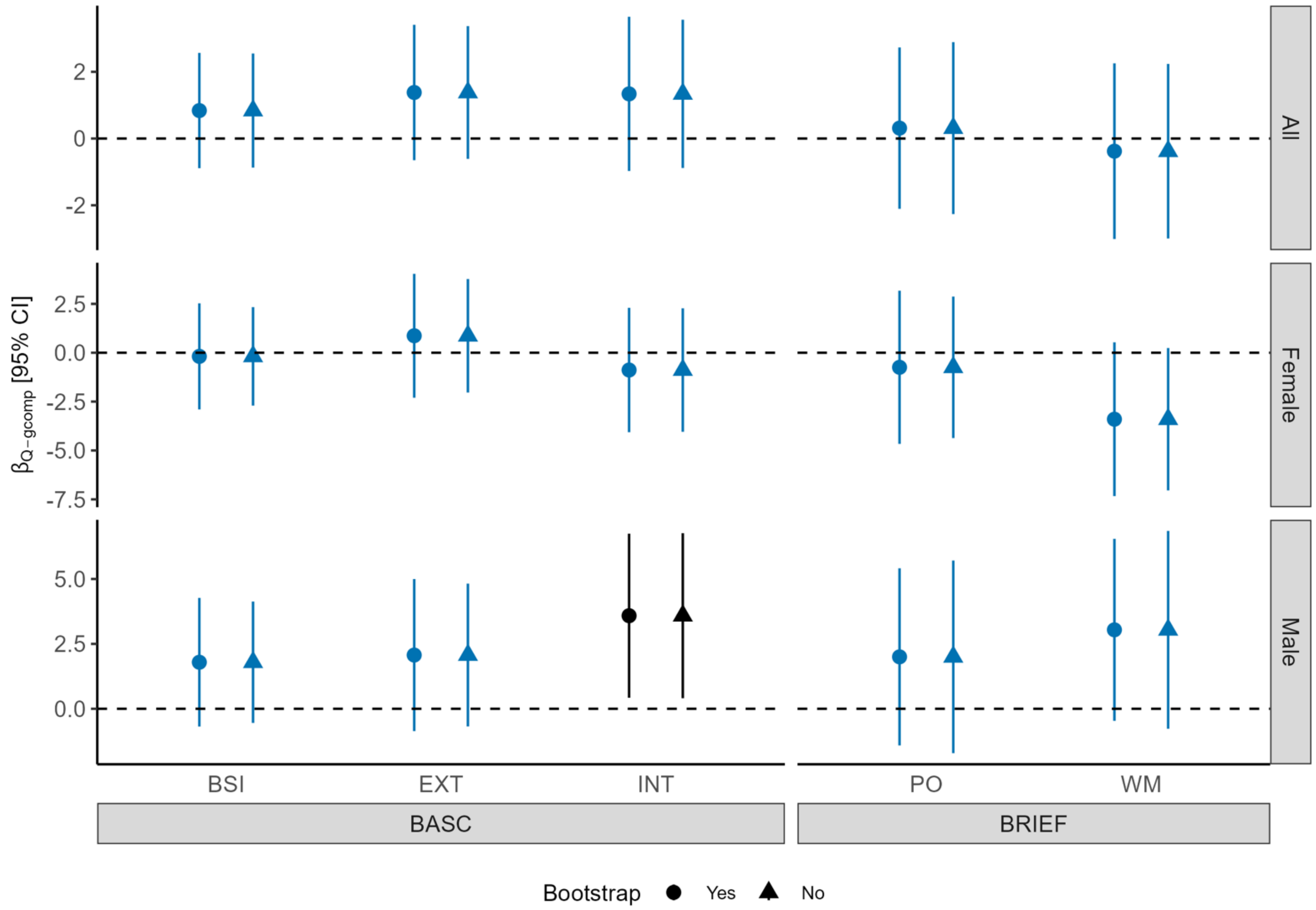

eFigure 5. Joint associations between gestational chemical biomarkers and child behavioral outcomes (n=695; Females=353, Males=342).

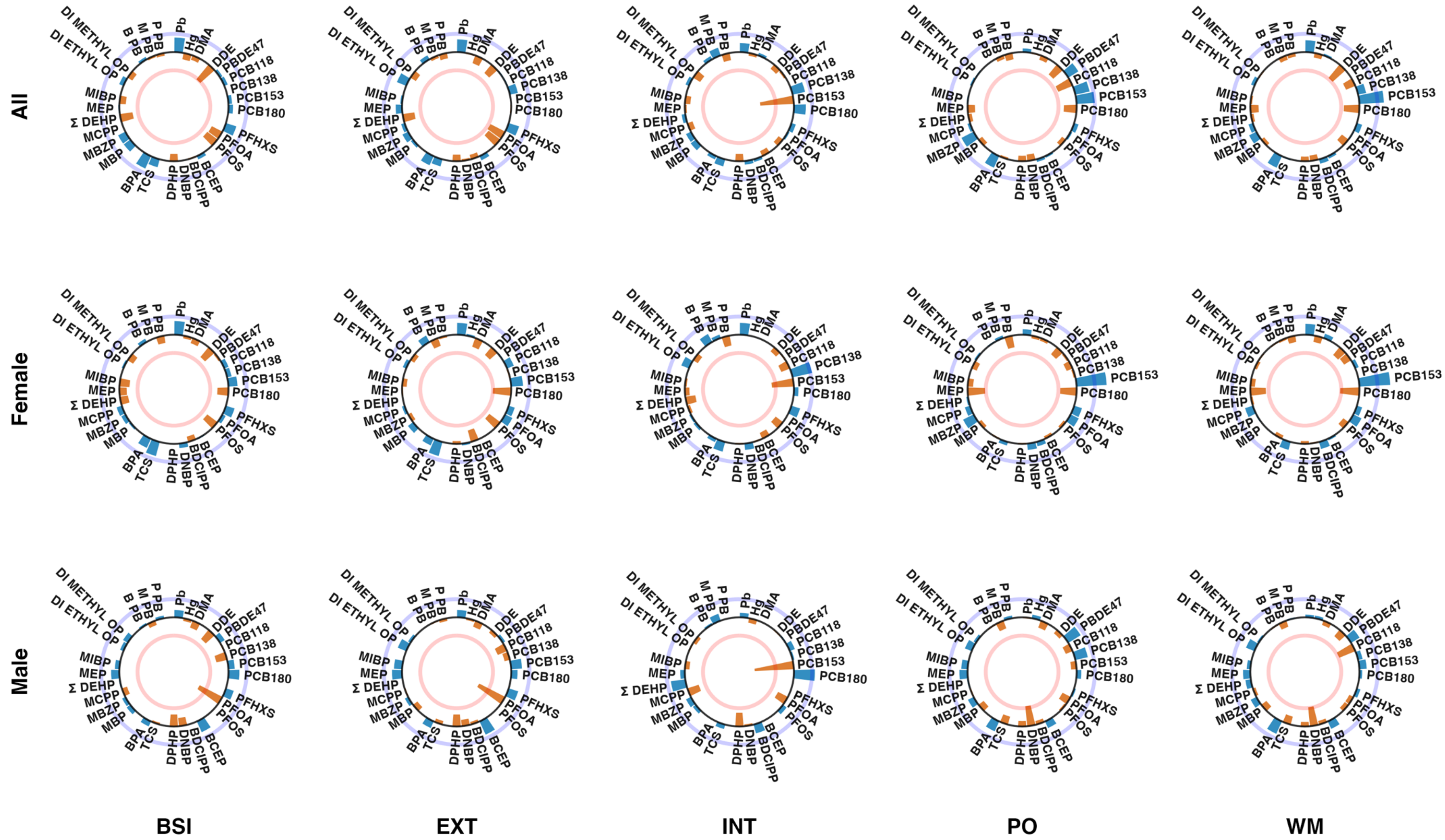

eFigure 6. Gestational chemical biomarker weights contributed to the joint associations (n=695; Females=353, Males=342).
Biomarker weights generated using quantile g-computation. Red circles represent a negative biomarker weight (-0.2), black represents null (zero), and the blue circle represent positive contributions (+0.2) to the joint associations.

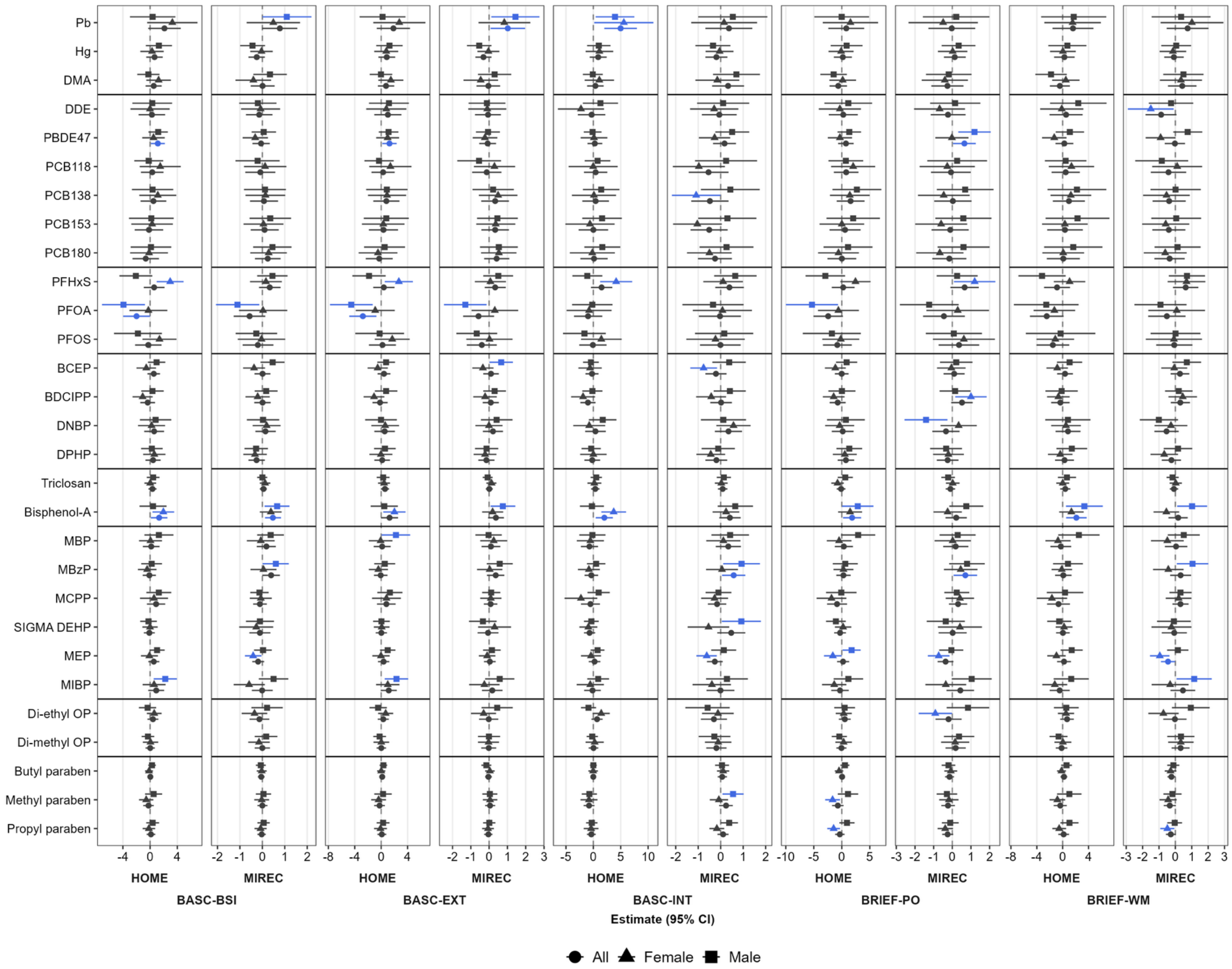

eFigure 7. Single gestational chemical biomarker associations with child behavioral outcomes (HOME study n= 139 [Males=66 & Females= 73]; MIREC n= 556 [Males=276 & Females= 280]).

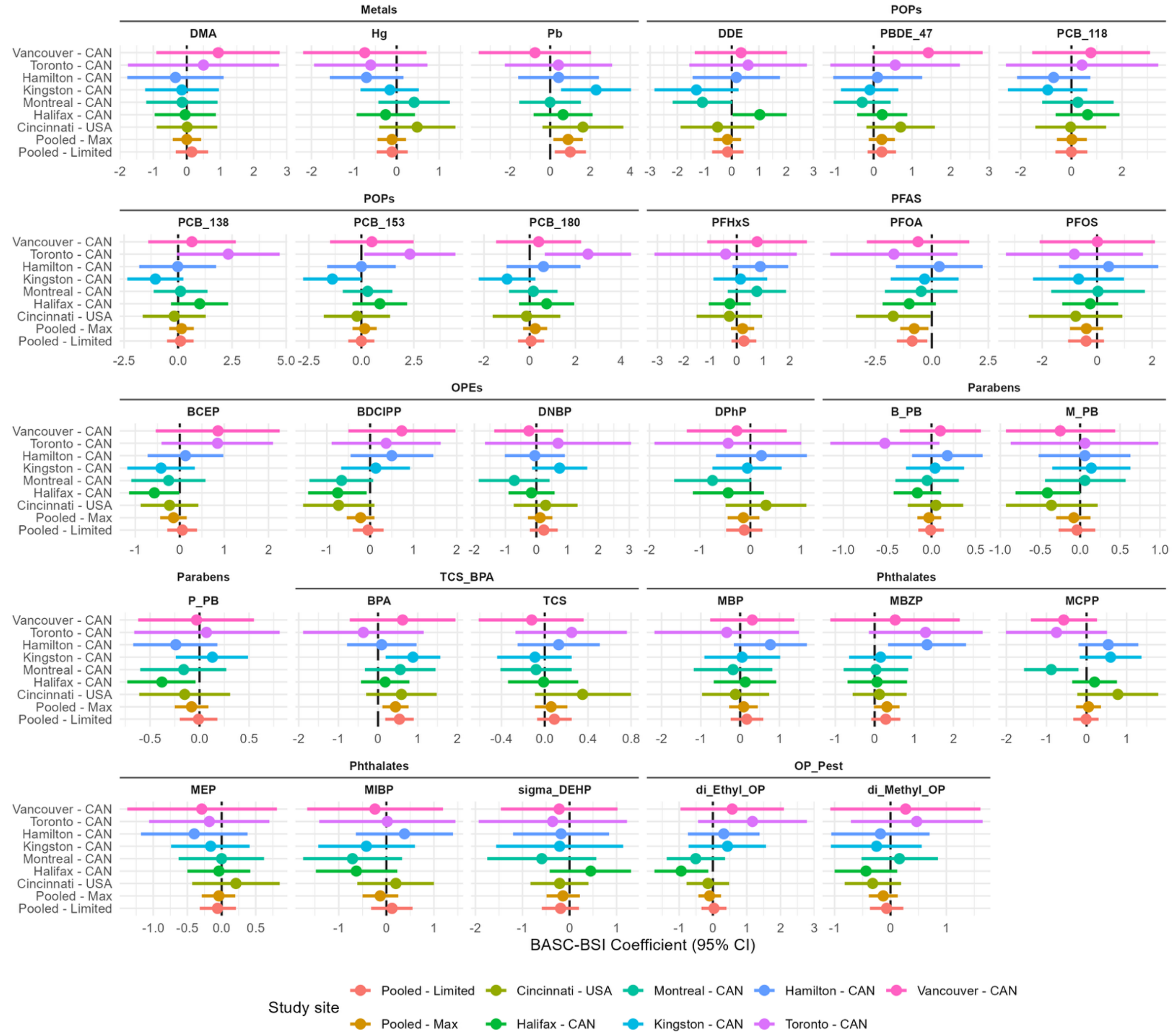

eFigure 8. Single gestational chemical biomarker associations with child behavioral symptom index using maximum study participants. The sample size by each exposure biomarker and outcome combination varies due to unmeasured exposure biomarkers. The sample size for pooled set vary between 844-894 participants (labeled as Pooled - Max). Whereas the Pooled – Limited includes 695 study participants with all 29 chemical biomarkers.

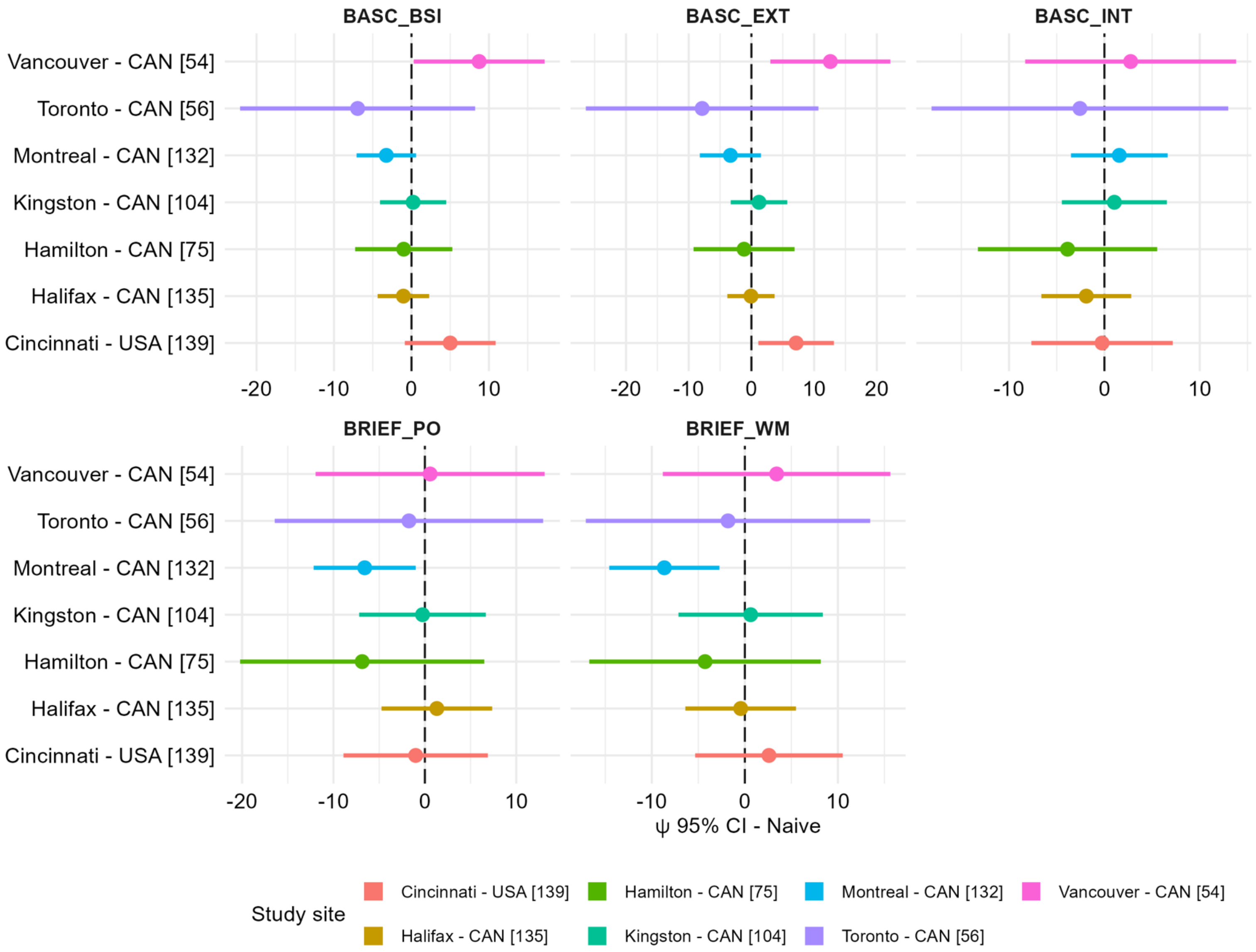

eFigure 9. Joint association between gestational chemical biomarkers and child behavioral outcomes by study sites.
Estimates and confidence intervals were calculated using the non-bootstrap version of the quantile g-computation regression.

eMethods 1. Biomarker Measurements

1.1 Metals

Lead (Pb) and total mercury (Hg) concentrations were measured in whole blood samples, and DMA concentrations were measured in urine samples. These concentrations were measured using inductively coupled plasma mass spectrometry (ICP-MS).[3-6] In the HOME Study, we measured DMA concentrations for 6% of participants using urine samples collected during the first study visit. We supplemented these measurements with data from the second visit, when we collected samples from 94% of participants at approximately 26 weeks of gestation.

1.2 Persistent Organic Pollutants (PBDEs and PCBs)

Concentrations of PBDE47 p,p'-dichlorodiphenyldichloroethylene (DDE), and PCB biomarkers from serum or plasma were measured using gas chromatography coupled with tandem mass spectroscopy (GC-MS).[7,8]

1.3 Perfluoroalkyl Substances (PFAS)

PFAS concentrations were measured using high-performance liquid chromatography-tandem mass spectroscopy (HPLC-MS) in the HOME Study[9] in serum and ultra-performance liquid chromatography with a mass spectrometer (UPLC-MS) in plasma from the MIREC Study[7].

1.4 Organophosphate Esters (OPEs)

In the HOME Study, urinary concentrations of OPEs were measured using solid-phase extraction and high-performance liquid chromatography-isotope dilution tandem mass spectrometry.[10,11] In the MIREC Study, these chemicals were measured using atmospheric pressure gas chromatography with a tandem quadrupole mass spectrometry system (APGC-MS/MS).[12]

1.5 Phenols, Phthalates, and Parabens

In the HOME Study, urinary biomarkers of phthalates, phenols, and parabens were measured using HPLC-MS.[13,14] In the MIREC study, these biomarkers were measured using UPLC-MS/MS.[15,16]

1.6 Organophosphate Insecticides

Urinary concentrations of dialkylphosphates were measured using GC-MS/MS.[17,18]

1.7 Urine Specific Gravity and Serum/Plasma Lipids

Urine specific gravity was measured using a handheld refractometer (Atago, Tokyo, Japan). In the MIREC Study, total lipids, including total cholesterol (TC), free cholesterol (FC), triglycerides (TG), and phospholipids (PL), were measured in plasma samples as grams per liter (g/L) using enzymatic techniques and colorimetric methods at the laboratory of the Centre Hospitalier de l'Université Laval. In the HOME Study, total lipids were calculated from triglyceride and total cholesterol measurements in serum samples using standard enzymatic methods.

1.8 Cotinine

In the HOME Study, serum cotinine (a biomarker of potential tobacco smoke exposure) was measured using HPLC-MS/MS.[19] In the MIREC Study, cotinine concentrations from plasma samples were measured using UPLC-MS/MS.[20]

The limits of detection (LOD) of biomarkers included from the HOME and MIREC Studies, along with the frequency of biomarker detection, are summarized in Table S1.

1. Woodruff TJ, Zota AR, Schwartz JM. Environmental chemicals in pregnant women in the United States: NHANES 2003-2004. *Environ Health Perspect*. Jun 2011;119(6):878–85. doi:10.1289/ehp.1002727
2. Oh J, Quirós-Alcalá L, Li X, et al. Prenatal exposure to organophosphate ester flame retardants and behavioral outcomes in early childhood in the Environmental influences on Child Health Outcomes (ECHO) cohort. *Environment International*. 2025/08/01/ 2025;202:109649. doi:https://doi.org/10.1016/j.envint.2025.109649
3. Nixon DE, Burritt MF, Moyer TP. The determination of mercury in whole blood and urine by inductively coupled plasma mass spectrometry. *Spectrochimica Acta Part B-Atomic Spectroscopy*. Aug 9 1999;54(8):1141–1153. doi:Doi 10.1016/S0584-8547(99)00057-9
4. Jones DR, Jarrett JM, Tevis DS, et al. Analysis of whole human blood for Pb, Cd, Hg, Se, and Mn by ICP-DRC-MS for biomonitoring and acute exposures. *Talanta*. Jan 1 2017;162:114–122. doi:10.1016/j.talanta.2016.09.060
5. Arbuckle TE, Liang CL, Morisset AS, et al. Maternal and fetal exposure to cadmium, lead, manganese and mercury: The MIREC study. *Chemosphere*. Nov 2016;163:270–282. doi:10.1016/j.chemosphere.2016.08.023
6. Ettinger AS, Arbuckle TE, Fisher M, et al. Arsenic levels among pregnant women and newborns in Canada: Results from the Maternal-Infant Research on Environmental Chemicals (MIREC) cohort. *Environ Res*. Feb 2017;153:8–16. doi:10.1016/j.envres.2016.11.008
7. Fisher M, Arbuckle TE, Liang CL, et al. Concentrations of persistent organic pollutants in maternal and cord blood from the maternal-infant research on environmental chemicals (MIREC) cohort study. *Environ Health*. May 4 2016;15(1):59. doi:10.1186/s12940-016-0143-y
8. Sjodin A, Jones RS, Lapeza CR, Focant JF, McGahee EE, 3rd, Patterson DG, Jr. Semiautomated high-throughput extraction and cleanup method for the measurement of polybrominated diphenyl ethers, polybrominated biphenyls, and polychlorinated biphenyls in human serum. *Anal Chem*. Apr 1 2004;76(7):1921–7. doi:10.1021/ac030381+
9. Kato K, Basden BJ, Needham LL, Calafat AM. Improved selectivity for the analysis of maternal serum and cord serum for polyfluoroalkyl chemicals. *J Chromatogr A*. Apr 15 2011;1218(15):2133–7. doi:10.1016/j.chroma.2010.10.051
10. Jayatilaka NK, Restrepo P, Davis Z, Vidal M, Calafat AM, Ospina M. Quantification of 16 urinary biomarkers of exposure to flame retardants, plasticizers, and organophosphate insecticides for biomonitoring studies. *Chemosphere*. Nov 2019;235:481–491. doi:10.1016/j.chemosphere.2019.06.181
11. Jayatilaka NK, Restrepo P, Williams L, Ospina M, Valentin-Blasini L, Calafat AM. Quantification of three chlorinated dialkyl phosphates, diphenyl phosphate, 2,3,4,5-tetrabromobenzoic acid, and four other organophosphates in human urine by solid phase extraction-high performance liquid chromatography-tandem mass spectrometry. *Anal Bioanal Chem*. Feb 2017;409(5):1323–1332. doi:10.1007/s00216-016-0061-4
12. Ashley-Martin J, MacPherson S, Zhao Z, et al. Descriptive analysis of organophosphate ester metabolites in a pan-Canadian pregnancy cohort. *Sci Total Environ*. Jul 20 2023;883:163327. doi:10.1016/j.scitotenv.2023.163327
13. Ye X, Bishop AM, Needham LL, Calafat AM. Automated on-line column-switching HPLC-MS/MS method with peak focusing for measuring parabens, triclosan, and other environmental phenols in human milk. *Anal Chim Acta*. Aug 1 2008;622(1-2):150–6. doi:10.1016/j.aca.2008.05.068

14. Silva MJ, Samandar E, Preau JL, Jr., Reidy JA, Needham LL, Calafat AM. Quantification of 22 phthalate metabolites in human urine. *J Chromatogr B Analyt Technol Biomed Life Sci*. Dec 1 2007;860(1):106–12. doi:10.1016/j.jchromb.2007.10.023
15. Arbuckle TE, Davis K, Marro L, et al. Phthalate and bisphenol A exposure among pregnant women in Canada--results from the MIREC study. *Environ Int*. Jul 2014;68:55–65. doi:10.1016/j.envint.2014.02.010
16. Arbuckle TE, Marro L, Davis K, et al. Exposure to free and conjugated forms of bisphenol A and triclosan among pregnant women in the MIREC cohort. *Environ Health Perspect*. Apr 2015;123(4):277–84. doi:10.1289/ehp.1408187
17. Sokoloff K, Fraser W, Arbuckle TE, et al. Determinants of urinary concentrations of dialkyl phosphates among pregnant women in Canada - Results from the MIREC study. *Environ Int*. Sep 2016;94:133–140. doi:10.1016/j.envint.2016.05.015
18. Bravo R, Caltabiano LM, Weerasekera G, et al. Measurement of dialkyl phosphate metabolites of organophosphorus pesticides in human urine using lyophilization with gas chromatography-tandem mass spectrometry and isotope dilution quantification. *J Expo Anal Environ Epidemiol*. May 2004;14(3):249–59. doi:10.1038/sj.jea.7500322
19. DeLorenze GN, Kharrazi M, Kaufman FL, Eskenazi B, Bernert JT. Exposure to environmental tobacco smoke in pregnant women: the association between self-report and serum cotinine. *Environ Res*. Sep 2002;90(1):21–32. doi:10.1006/enrs.2001.4380
20. Arbuckle TE, Liang CL, Fisher M, Caron NJ, Fraser WD, and the MSG. Exposure to tobacco smoke and validation of smoking status during pregnancy in the MIREC study. *J Expo Sci Environ Epidemiol*. Sep 2018;28(5):461–469. doi:10.1038/s41370-017-0011-z
